# Lake Poso's shrimp fauna revisited: the description of five new species of the genus *Caridina* (Crustacea, Decapoda, Atyidae) more than doubles the number of endemic lacustrine species

**DOI:** 10.3897/zookeys.1009.54303

**Published:** 2021-01-04

**Authors:** Werner Klotz, Thomas von Rintelen, Daisy Wowor, Chris Lukhaup, Kristina von Rintelen

**Affiliations:** 1 Wiesenweg 1, A-6063 Rum, Austria Unaffiliated Rum Austria; 2 Museum für Naturkunde, Leibniz Institute for Evolution and Biodiversity Science, Invalidenstr. 43, D-10115 Berlin, Germany Leibniz Institute for Evolution and Biodiversity Science Berlin Germany; 3 Division of Zoology, Research Center for Biology, Indonesian Institute of Sciences (LIPI), Jalan Raya Jakarta Bogor Km 46, Cibinong 16911, Indonesia Research Center for Biology, Indonesian Institute of Sciences Cibinong Indonesia; 4 Waldstrasse 5a, D-66999 Hinterweidenthal, Germany Unaffiliated Hinterweidenthal Germany

**Keywords:** Adaptive radiation, ancient lake, freshwater biodiversity, Indonesia, integrative taxonomy, Sulawesi

## Abstract

Lake Poso, an ancient lake system on the Indonesian island Sulawesi, harbours an endemic species flock of six, four lacustrine and two riverine species of the freshwater shrimp genus *Caridina*. In this study, five new lacustrine species are described, bringing the total to eleven species altogether. The number of lacustrine species is more than doubled to nine species compared to the last taxonomic revision in 2009. One of them, *Caridina mayamareenae* Klotz, Wowor & von Rintelen, **sp. nov**., even represents the first case of an atyid shrimp associated with freshwater snails which is morphologically adapted to living in shells. An integrative approach was used by providing a combination of morphological, ecological, and molecular data. Based on standard morphological characters, distribution, substrate preferences, and colouration of living specimens in the field, five distinct undescribed species could be distinguished. To support our species-hypothesis based on the mitochondrial genes 16S and COI, a molecular phylogeny was used for all eleven species from Lake Poso. All species form a well-supported monophyletic group, but only four morphospecies consistently correspond to mtDNA clades – a possible reason could be introgressive hybridisation, incomplete lineage sorting, or not yet fixed species boundaries. These results are discussed further in the context of adaptive radiation, which turned out to be more diverse than previously described. Finally, yet importantly, subjecting all new species to similar threats and to the same IUCN category and criterion than the previously described species from the lake is recommended.

## Introduction

**Figure 1.**
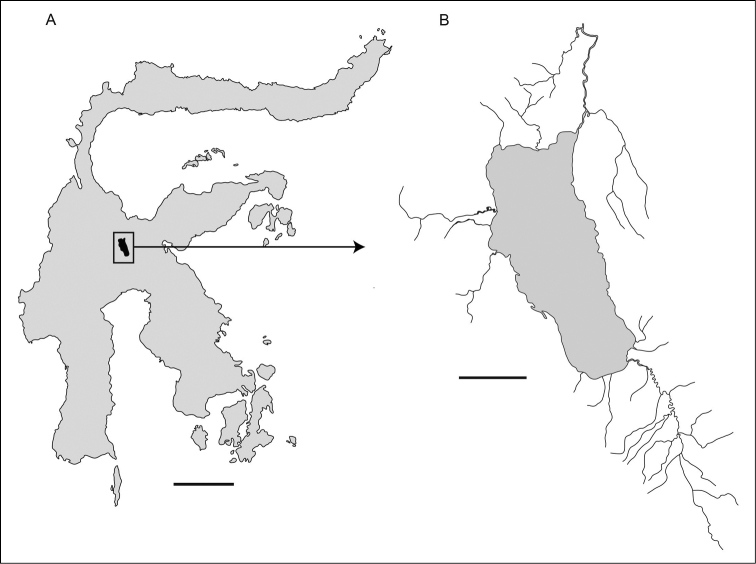
Lake Poso situated in the central highlands of the Indonesian island Sulawesi **A** Sulawesi (scale bar: 100 km) **B** Lake Poso and catchment area (scale bar: 10 km). Map modified from von Rintelen et al. 2007a.

Lake Poso (Fig. 1) is one of the two so-called ancient lakes systems on the Indonesian island of Sulawesi. This long-lived lake probably is more than 1 million years old (Vaillant et al. 2011) and regarded as a hotspot of biodiversity (von Rintelen et al. 2012). The lake is of tectonic origin and has an area of 323.2 km^2^, maximum depths of 450 m, is oligotrophic with a high transparency and low organic content (von Rintelen et al. 2012). It provides ideal conditions for the evolution of highly diverse endemic species flocks of freshwater organisms such as crustaceans, molluscs and fishes (see review in von Rintelen et al. 2012).

The endemic species flock of atyid freshwater shrimps of the genus *Caridina* in Lake Poso was first studied by Schenkel (1902) with the description of two new species from the lake itself and one riverine species from the lake's catchment. More than 100 years later, another lacustrine species was described by Cai and Wowor (2007), followed by von Rintelen and Cai (2009), who revised the entire species flock of four lacustrine and two riverine species in the lake system including the description of a new lacustrine and a new riverine species (Table 1).

Here, we use an integrative taxonomic approach to study newly collected material from Lake Poso to a) discover new, so far unknown species from the lake, b) provide a combination of morphological, ecological, and molecular data to describe the newly discovered species, c) provide two different identification keys (a regular key for preserved specimens and a key for pre-sorting living specimens in the field without having to use a microscope), and d) discuss the results in context of adaptive radiation and conservation status of the previously revised Lake Poso species flock.

## Materials and methods

Specimens were caught by hand net and preserved in 75–95% ethanol during several fieldtrips to Lake Poso (Fig. 1) between 2003 and 2019 (for sampling details, see Systematic accounts). Specimens were dissected and morphometrical data were taken using a BMS 143 Trino Zoom dissecting microscope with an ocular grid. Details on setae and mouthparts were observed using a Reichert Biovar compound microscope. Rostral characters were taken from all specimens examined. Drawings were made from microphotographs using Adobe Illustrator following Coleman (2003, 2006). The setae terminology used mostly follows Short (2004). The two identification keys in this study were modified and updated from the previous keys in von Rintelen and Cai (2009). All new species were described by the first, third and last author.

All material examined is deposited in Museum Zoologicum Bogoriense, Cibinong, Indonesia (**MZB**), and the Museum für Naturkunde (Museum of Natural History), Berlin, Germany (**ZMB**). The following abbreviations are used in the text: **cl.**, carapace length (measured from the postorbital margin to the posterior margin of the carapace); **ov**., ovigerous; **E**, east; **S**, south; **N**, north; **W**, west.

DNA was extracted from abdominal tissue using either a Qiagen Blood and Tissue Kit or a Qiagen BioSprint with the Plant Kit (but lysis with 10ml Qiagen Proteinase K (20mg/ml) added) according to the manufacturer's instructions. Fragments of the mitochondrial 16S rRNA (16S, ~ 590 bp) and cytochrome oxidase subunit I (COI, 861 bp) genes were amplified by polymerase chain reaction (PCR) and sequenced using primers 16S-F-Car and 16S-R-Car1 (16S), and COI-F-Car and COI-R-Car (COI) (von Rintelen et al. 2007a), or, for COI only, COI-F-Car and COI-R-H16mod3 (1087 bp fragment extending COI-F-Car/COI-R-Car fragment at 3’ end; 5’ CAAYKATCTGCCATTTTAGA), sometimes in combination with COI-F-Car and COI-R-int (458 bp fragment at 5’ end of COI-F-Car/COI-R-Car fragment; 5’ GCAATAATTATAGTTGCTGA). In the latter case, sequencing was done using COI-R-int and COI-R-H16mod3. Amplifications were conducted in 25 µL volumes containing 50–100 ng DNA, 1x PCR buffer, 200 mM of each dNTP, 0.5 mM of each primer, 2 mM MgCl2 and 1 U of Taq polymerase. After an initial denaturation step of 3 min at 94 °C, 35 cycles of 30 sec at 94 °C, 60 sec at 45°C (COI) or 50°C (16S) and 60 sec (16S) or 90 sec (COI) at 72 °C were performed, followed by a final extension step of 5 min at 72 °C. PCR products were sent to Macrogen Europe for purification and sequencing of both strands of the amplified gene fragments using the primers as given above.

Contigs of forward and reverse strands were assembled using Geneious Prime (v. 2019.2.1) and corrected by eye. Sequences were aligned by eye (COI) and with MAFFT (16S) (Katoh and Standley 2013). To determine the best substitution model for Bayesian inference analyses (see below), hierarchical likelihood ratio tests were carried out with jModelTest (Posada 2008) on both sequence sets. Based on the Akaike Inference Criterion (AIC), the GTR + I + G (COI) and the HKY + I + G (16S) models were chosen. The datasets were analysed further concatenated.

All new sequences (51 from Lake Poso species, 1 outgroup taxon) have been deposited in GenBank (for accession numbers and museum voucher numbers see Suppl. material 1, Table S1). Additionally, the sequences of Lake Poso species of *Caridina* published by von Rintelen et al. (2007a) have been included in the analysis and sequences from two endemic outgroup taxa from Sulawesi published in von Rintelen et al. (2010) (Suppl. material 1, Table S1).

Phylogenetic trees were reconstructed by Bayesian inference (BI; Huelsenbeck et al. 2001) using MrBayes 3.2.6 (Ronquist et al. 2012). The MCMCMC-algorithm was run with four independent chains for 20,000,000 generations, samplefreq = 500, and burnin = 25%. Maximum likelihood analyses were run with IQ-TREE (Nguyen et al. 2015) and branch support was obtained through the implemented ultrafast bootstrap (1,000 replicates; Hoang et al. 2018). BI and ML analyses were run using two gene partitions with the models specified above (for IQ-TREE, see Chernomor et al. 2016). In addition, Maximum Parsimony (MP) analyses were performed using the heuristic search algorithm as implemented in PAUP* (Swofford 2002), with gaps treated as fifth base. Support for nodes was estimated by bootstrap analysis (1,000 fast stepwise-addition bootstrap replicates). Genetic distances were calculated using MEGA X (Kumar et al. 2018).

## Results

**Figure 2.**
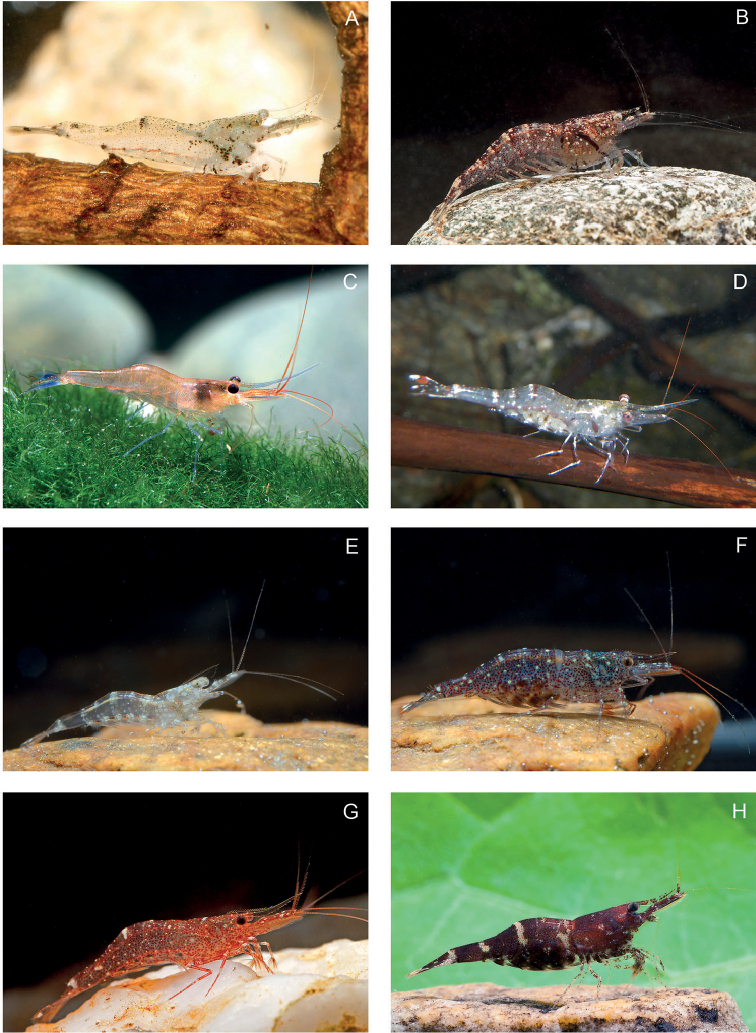
Living specimens of *Caridina* spp in Lake Poso 1. **A***Caridina schenkeli* von Rintelen & Cai, 2009 **B***C. acutirostris* Schenkel, 1902 **C***C. caerulea* von Rintelen & Cai, 2009 **D***C. ensifera* Schenkel, 1902 **E***C. mayamareenae* sp. nov. male **F, G***C. longidigita* Cai & Wowor, 2007 **H***C. sarasinorum* Schenkel, 1902. Not to scale. Photo: C. Lukhaup (**A, C**), W. Klotz (**B, D–H**).

**Figure 3.**
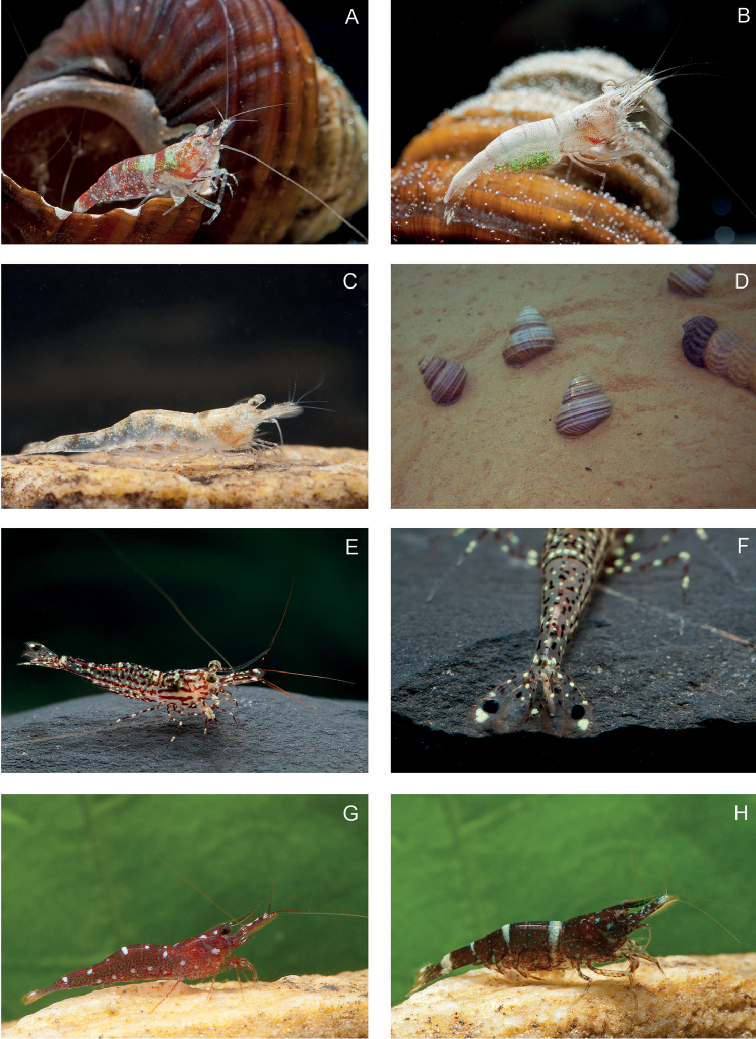
Living specimens of *Caridina* spp in Lake Poso 2. **A, B***Caridina mayamareenae* sp. nov. **C***C. lilianae* sp. nov. **D** Two snail species *Celetaia persculpta* and *Tylomelania* sp. on soft substrate. Empty shells of these species are shelter for *C. mayamareenae* sp. nov. **E, F***C. poso* sp. nov. **G***C. marlenae* sp. nov. **H***C. fusca* sp. nov. Not to scale. All photographs: W. Klotz.

We distinguished five morphologically distinct and undescribed species that could be separated clearly based on the examination of living specimens in the field (Figs 2E, 3A–C, E–H) and preserved in ethanol that turns specimens completely colourless. In Lake Poso, there are eleven species altogether, comprising a species flock of nine lacustrine and two riverine species (Table 1). This more than doubles the previously known lacustrine fauna of four species (von Rintelen and Cai 2009). Distribution data limited to Lake Poso and reproductive biology (few (5–36), large-sized eggs ca. 0.7–1.1 mm length of developed eggs with eyespots) indicative of direct larval development (Lai and Shy 2009) suggest endemism of all Lake Poso species (see Systematic accounts of this study; von Rintelen and Cai 2009).

**Table 1.** Current checklist of endemic species of the genus *Caridina* from Lake Poso, Indonesia.

Species	Remarks	Reference
*Caridina acutirostris* Schenkel, 1902	Exclusively riverine species, endemic to Lake Poso catchment	von Rintelen and Cai 2009
*C. caerulea* von Rintelen & Cai, 2009	Endemic to Lake Poso (excluding rivers)	von Rintelen and Cai 2009
*C. ensifera* Schenkel, 1902	Endemic to Lake Poso (excluding rivers)	von Rintelen and Cai 2009
*C. fusca* Klotz, Wowor & von Rintelen, sp. nov.	Endemic to Lake Poso (excluding rivers)	This study
*C. lilianae* Klotz, Wowor & von Rintelen, sp. nov.	Endemic to Lake Poso (excluding rivers)	This study
*C. longidigita* Cai & Wowor, 2007	Endemic to Lake Poso (excluding rivers)	von Rintelen and Cai 2009
*C. marlenae* Klotz, Wowor & von Rintelen, sp. nov.	Endemic to Lake Poso (excluding rivers)	This study
*C. mayamareenae* Klotz, Wowor & von Rintelen, sp. nov.	Endemic to Lake Poso (excluding rivers); hiding in empty snail shells	This study
*C. poso* Klotz, Wowor & von Rintelen, sp. nov.	Endemic to Lake Poso (excluding rivers)	This study
*C. sarasinorum* Schenkel, 1902	Endemic to Lake Poso (excluding rivers)	von Rintelen and Cai 2009
*C. schenkeli* von Rintelen & Cai, 2009	Exclusively riverine species, endemic to Lake Poso catchment	von Rintelen and Cai 2009

### Identification key to species of the genus *Caridina* from Lake Poso system

**Table:** 

1	Tip of rostrum reaching end to distinctly overreaching end of scaphocerite (Fig. 4A, C)	**2**
–	Tip of rostrum not reaching end of scaphocerite (Fig. 4B)	**8**
2	Tip of rostrum reaching or slightly overreaching end of scaphocerite (Fig. 4C)	**3**
–	Tip of rostrum reaching distinctly beyond end of scaphocerite (Fig. 4A)	**4**
3	Epipods present on first and second pereiopods (Fig. 4D)	***C. schenkeli* von Rintelen & Cai, 2009**
–	Epipod absent from all pereiopods (Fig. 4E)	***C. marlenae* sp. nov**.
4	Tip of rostrum reaching beyond end of scaphocerite, ~ 0.9–1.4 times as long as carapace, long but not very slender (Fig. 4F)	**5**
–	Tip of rostrum reaching far beyond end of scaphocerite, ~ 1.4–2.8 times as long as carapace, long and quite slender (Fig. 4A)	**6**
5	Epipod present on first pereiopod; chelae of first and second pereiopods stout, setae on tip of fingers ~ half as long as chelae (Fig. 4G)	***C. sarasinorum* Schenkel, 1902**
–	Epipod absent from all pereiopods; chelae of first and second pereiopods very slender, setae on tip of fingers ~ as long as chelae (Fig. 4H)	***C. longidigita* Cai & Wowor, 2007**
6	Epipod absent from all pereiopods, vestigial epipod present on third maxilliped (Fig. 4I)	***C. poso* sp. nov**.
–	Epipods present on third maxilliped, first and second pereiopods (Fig. 4D)	**7**
7	Rostrum 1.4–2.3 times as long as carapace, with distinctly less teeth (dorsal 9–15, ventral 16–29) (Fig. 4J); uropodal diaeresis with 9–11 spiniform setae; dactylus of third pereiopod with 6–9 spiniform setae; dactylus of fifth pereiopod with 51–57 serrate setae	***C. ensifera* Schenkel, 1902**
–	Rostrum 1.9–2.6 times as long as carapace, with distinctly more teeth (dorsal 11–20, ventral 26–48) (Fig. 4K); uropodal diaeresis with 11–14 spiniform setae; dactylus of third pereiopod with 4–5 spiniform setae; dactylus of fifth pereiopod with 27–49 serrate setae	***C. caerulea* von Rintelen & Cai, 2009**
8	Rostrum very short, tip not reaching distal margin of eye (Fig. 7A, B)	***C. lilianae* sp. nov**.
–	Rostrum moderately short, tip distinctly overreaching distal margin of eye	**9**
9	Tip of rostrum reaching end of third segment of antennular peduncle (Fig. 5A)	***C. fusca* sp. nov**.
–	Tip of rostrum reaching end of second segment of antennular peduncle (Fig. 4L)	**10**
10	Rostrum high, maximum depth of rostrum more than maximum dorsoventral diameter of eye, ~ 0.17 of dorsal margin of rostrum distal without tooth (Fig. 11A, B)	***C. mayamareenae* sp. nov**.
–	Rostrum slender, maximum depth of rostrum less than maximum dorsoventral diameter of eye, ~ 0.3–0.5 of dorsal margin of rostrum distal without tooth (Fig. 4L)	***C. acutirostris* Schenkel, 1902**

**Figure 4.**
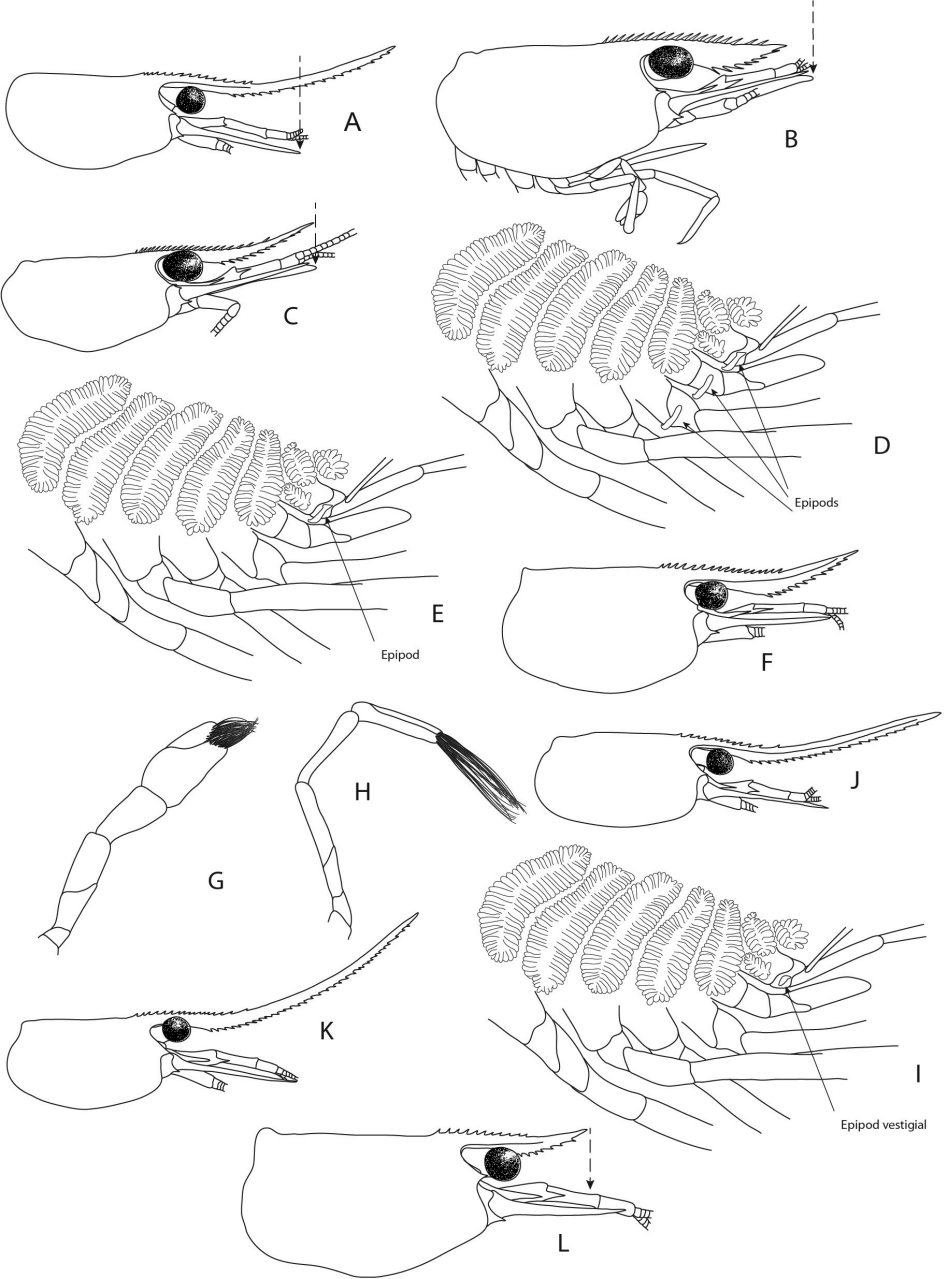
Morphological characters used in the identification keys **A** tip of rostrum distinctly overreaching end of scaphocerite **B** tip of rostrum not reaching end of scaphocerite **C** tip of rostrum reaching end of scaphocerite **D** epipods present on third maxilliped and first and second pereiopods **E** epipod present on third maxilliped but absent from all pereiopods **F** rostrum long but not very slender **G** chelae of first and second pereiopods stout, setae on tip of fingers approx. half as long as chelae **H** chelae of first and second pereiopods very slender, setae on tip of fingers approx. as long as chelae **I** epipod vestigial present on third maxilliped and absent from all pereiopods **J** rostrum of *C. ensifera* Schenkel, 1902 **K** rostrum of *C. caerulea* von Rintelen & Cai, 2009 **L** rostrum reaching end of second segment of antennular peduncle and slender.

### Key for pre-sorting living *Caridina* in the field (Lake Poso system)^1^

**Table:** 

1	Shrimps collected from the rivers	2
–	Shrimps collected from the lake	3
2	Rostrum approximately as long (0.9–1.1 times) as carapace; body transparently yellowish or brownish (Fig. 2A)	*C. schenkeli* von Rintelen & Cai, 2009
–	Rostrum always distinctly shorter (0.3–0.7 times) than carapace; body transparently yellowish or brownish (Fig. 2B)	*C. acutirostris* Schenkel, 1902
3	Rostrum distinctly long and very slender, bent upwards, tip reaching far beyond end of scaphocerite; body rather slender and mainly transparent or with mottled pattern	4
–	Rostrum short to moderately long and not conspicuously slender, tip slightly reaching beyond end of scaphocerite or shorter; body usually more robust and less transparent	6
4	Body showing a clearly visible red-and-white stripe pattern (Fig. 3E); tail fan with black-and-white blotches (Fig. 3F)	*C. poso* sp. nov.
–	Body rather yellowish-transparent without stripe pattern; tail fan with colour marks	5
5	Legs and rostrum bluish; tail fan with two conspicuous blue patches (Fig. 2C)	*C. caerulea* von Rintelen & Cai, 2009
–	Legs and rostrum yellowish-reddish; tail fan with two conspicuous red patches (Fig. 2D)	*C. ensifera* Schenkel, 1902
6	Rostrum conspicuously high; large females whitish, frequently with broad red stripes and blotches, eggs green (Fig. 3A, B), males mostly transparent with some white blotches (Fig. 2E); living in empty snail shells	*C. mayamareenae* sp. nov.
–	Rostrum not conspicuously high; usually not found in empty snail shells	7
7	Rostrum very short, tip not reaching distal margin of eyes and body transparent-whitish (Fig. 3C); lives on very fine sand or soil in shallow water (1.5–2.5m)	*C. lilianae* sp. nov.
–	Rostrum distinctly longer, clearly overreaching distal margin of eyes; lives on various types of substrates	8
8	Chelae on first two pairs of pereiopods with very long and clearly visible fingers, setae on tip of fingers as long as or longer than chelae (Fig. 2F, G, 4H)	*C. longidigita* Cai & Wowor, 2007
–	Chelae on first two pairs of pereiopods not conspicuously long with rather short fingers, setae on tip of fingers shorter than chelae (Fig. 4G)	9
9	Body bright reddish with large white dots (Fig. 3G)	*C. marlenae* sp. nov.
–	Body dark reddish or brown with well-defined white transversal bands	10
10	Body dark reddish or brown with sharply defined white transversal bands on first, third, fifth and sixth abdominal segments (Fig. 3H); found under rocks	*C. fusca* sp. nov.
–	Body with a similar colouration and pattern, but bands less well-defined and scraggy (Fig. 2H); habitat not restricted to rocks	*C. sarasinorum* Schenkel, 1902

### Systematic accounts

#### Atyidae De Haan, 1849

***Caridina* H. Milne Edwards, 1837**

##### Caridina fusca

Taxon classificationAnimaliaDecapodaAtyidae

Klotz, Wowor & K. von Rintelen sp. nov.

D12AFA09-BC83-5962-B40C-1CB846E9BABB

http://zoobank.org/C6EF012A-7452-4C07-9E21-0FC6B2BC3082

Figures 3H , 5 , 6

###### Material examined.

**Holotype**: ov. ♀ cl. 2.9 mm (MZB Cru 5031), Indonesia, Central Sulawesi, Lake Poso, E shore, S of Tentena, dive at small cape, 15 m, 1°46.39'S, 120°38.33'E, M. Glaubrecht and T. von Rintelen leg., 12 May 2007. **Paratypes**: 1 ov. ♀ cl. 2.7 mm, 1 ♂ cl. 2.3 mm (MZB Cru 5032), 2 ♀♀ cl. 2.9 and 3.1 mm (ZMB 29518), same data as holotype; 1 ov. ♀ cl. 2.8 mm (MZB Cru 5033), Lake Poso, E shore, S of Tentena, dive at small cape, 15 m, 1°46.394'S, 120°38.327'E, coll. J. Pfaender and T. von Rintelen leg., 21 Sep. 2015; 1 ♀ cl. 2.4 mm (ZMB 29622), Lake Poso, E shore, S of Tentena, dive at small cape, 10 m, 1°46.394'S, 120°38.327'E, coll. J. Pfaender and T. von Rintelen leg., 21 Sep. 2015; 1 ov. ♀ cl. 2.7 mm (MZB Cru 5034), 1 ♀ cl. 2.5 mm (ZMB 30223), Lake Poso, E shore, S of Tentena, dive at small cape, 15 m, 1°46.394'S, 120°38.327'E, coll. T. von Rintelen and W. Klotz leg., 12 May 2017; 1 ov. ♀ cl. 2.9 mm (ZMB 30715), Lake Poso, W shore, Bay S of cape, in ca. 6 m depth, 1°55.408'S, 120°33.315'E, coll. T. von Rintelen leg., 4 Jul. 2018. 1 ov. ♀ cl. 2.4 mm (MZB Cru 5090), Lake Poso, E shore, small bay within mouth of outlet, 1°46.30'S, 120°38.38'E, T. von Rintelen leg., 14 Jul 2019.

###### Description.

**Figure 5.**
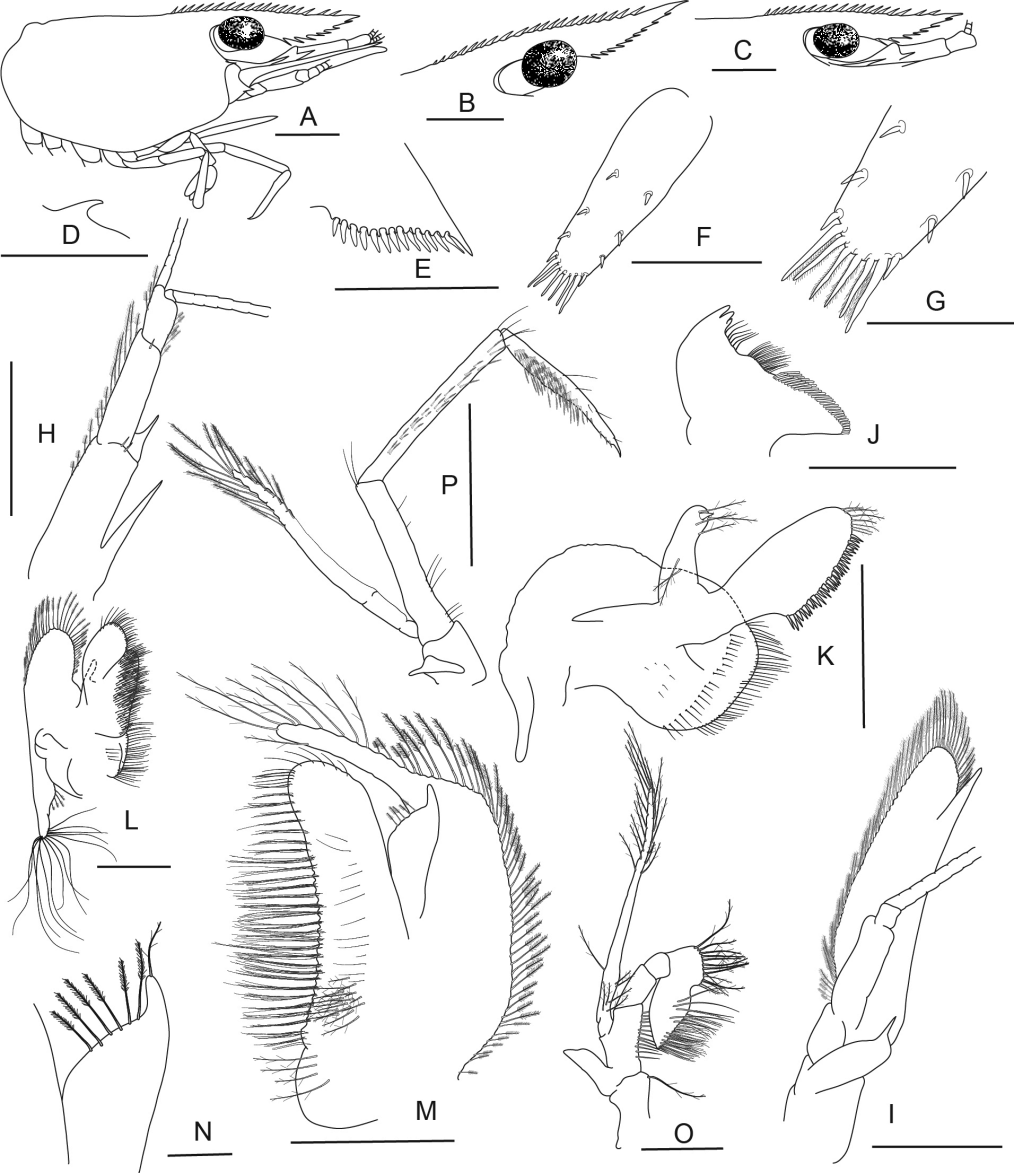
*Caridina fusca* sp. nov. Morphology 1. Paratype ov. ♀, cl. 2.7 mm, MZB Cru 5034 **A** cephalothorax and cephalic appendages **C** rostrum **D** preanal carina **E** uropodal diaeresis **F, G** telson **I** scaphocerite **J** distal part of mandible **K** maxillula **M** first maxilliped **N** Palp of first maxilliped **O** second maxilliped **P** third maxilliped; paratype ov. ♀, cl. 2.7 mm, MZB Cru 5032 **B** rostrum **H** antennular peduncle **L** maxilla. Scale bars: 1 mm (**A–C, F, H, I**); 0.5 mm (**D, E, G, J–M, O**); 0.1 mm (**N**).

***Cephalothorax and cephalic appendages*.** Postorbital carapace length 2.4–2.8 mm (n = 10). Rostrum (Fig. 5A–C) moderately long, straight or slightly sigmoid, reaching to end of antennular peduncle, dorsal and ventral margin armed throughout almost to tip, 0.78–0.94 (median 0.87, n = 7) times as long as carapace, rostral formula 5–7 + 12–17 / 7–9. Antennal spine fused with or slightly separated from orbital margin. Pterygostomial angle broadly rounded. Eyes well developed with globular cornea. Antennular peduncle (Fig. 5A, C, H), 0.84–0.90 (median 0.86, n = 4) times as long as carapace, first segment 1.71–2.14 (median 1.81, n = 4) times as long as second segment, second segment 2.33–3.00 (median 2.83, n = 4) times longer than third segment. Tooth on distolateral margin of first segment of antennular peduncle acute. Stylocerite reaching to 0.85–0.89 (median 0.87, n = 4) of first segment of antennular peduncle. Scaphocerite (Fig. 5I) 3.81–4.00 (median 3.90, n = 2) times as long as wide, inner and distal margin beset with plumose setae.

**Abdominal somites, telson, and uropods.** Sixth abdominal somite 0.54–0.58 (median 0.56, n = 4) times carapace length, 1.53–2.21 (median 1.77, n = 4) times as long as fifth somite, 0.94–1.00 (median 0.98, n = 4) times as long as telson. Distal margin of telson (Fig. 5F, G) convex without a median projection, with three pairs of short spiniform setae dorsally and one pair of short spiniform setae dorsolaterally; distal end with six long spiniform setae, lateral pair longer than others. Preanal carina (Fig. 5D) with a hook-like spine. Uropodal diaeresis (Fig. 5E) with 11–13 short movable spiniform setae, outermost ones shorter than lateral angle.

***Mouthparts and branchiae*.** Incisor process of mandible (Fig. 5J) ending in irregular teeth, molar process truncated. Lower lacinia of maxillula (Fig. 5K) broadly rounded, upper lacinia elongate, with numerous distinct teeth on inner margin, palp slender with few pappose setae and one conical spiniform seta near tip. Upper endites of maxilla (Fig. 5L) subdivided, palp slender, scaphognathite tapering posteriorly, fringed with long, curved setae at posterior margin. Palp of first maxilliped (Fig. 5M, N) ending in a slender triangular extension. Podobranch on second maxilliped (Fig. 5O) reduced to a lamina. Third maxilliped (Fig. 5P) with one well developed and one small arthrobranch, ultimate segment of maxilliped shorter than penultimate segment. First pereiopod with a small arthrobranch. Pleurobranchs present on all pereiopods. Epipod slightly reduced (without distal hook) on third maxilliped, absent from all pereiopods.

**Figure 6.**
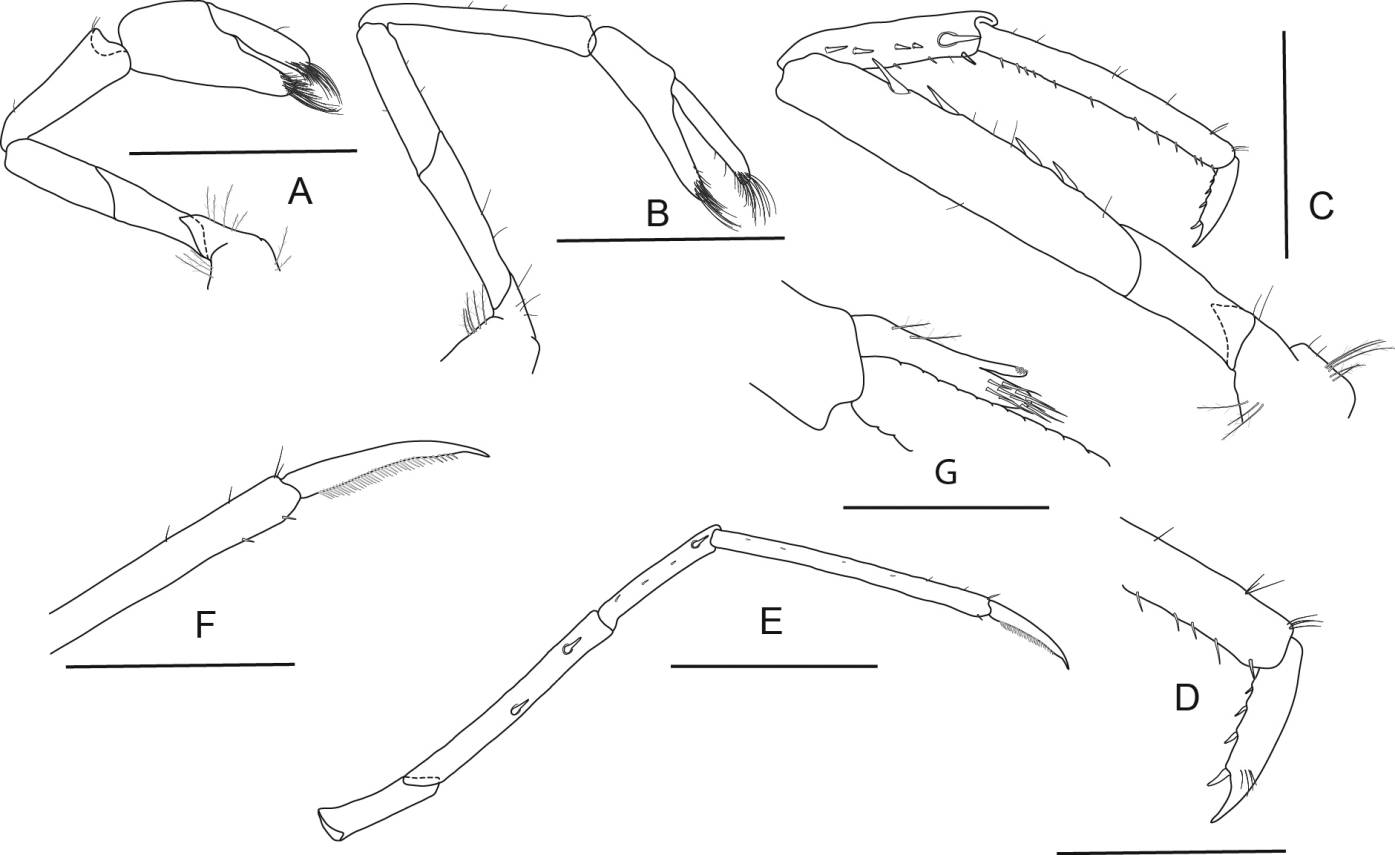
*Caridina fusca* sp. nov. Morphology 2. Paratype ov. ♀, cl. 2.7 mm, ZMB 30223 **A** first pereiopod **B** second pereiopod **C** third pereiopod **D** dactylus of third pereiopod; paratype ov. ♀, cl. 2.7 mm, ZMB 29518 **E** fifth pereiopod **F** dactylus of fifth pereiopod; paratype ♂, cl. 2.3 mm, MZB Cru 5032 **G** appendix masculina on male second pleopod. Scale bars: 1 mm (**A–C, E**); 0.5 mm (**D, F, G**).

***Pereiopods*.** Chelae of first and second pereiopods (Fig. 6A, B) well developed; chela of first pereiopod 2.29–2.73 (median 2.33, n = 7) times as long as wide, 1.17–1.34 (median 1.31, n = 7) times length of carpus; tips of fingers rounded, without hooks, with tufts of hairs near tip; dactylus 0.94–1.21 (median 1.11, n = 7) times as long as palm; carpus slender, hardly excavated distally, 2.33–3.00 (median 2.58, n = 7) times as long as wide, 1.21–1.32 (median 1.29, n = 5) times length of merus. Merus 2.32–3.43 (median 2.83, n = 5) times as long as wide, 0.85–0.96 (median 0.86, n = 4) times as long as ischium. Chela of second pereiopod 2.70–3.94 (median 3.31, n = 4) times as long as wide, 0.62–0.87 (median 0.74, n = 7) times length of carpus; tips of fingers rounded, without hooks, with tufts of hairs near tip; dactylus 1.11–1.50 (median 1.21, n = 7) times as long as palm; carpus 4.89–7.17 (median 6.14, n = 7) times as long as wide, 1.33–1.51 (median 1.46, n = 7) times as long as merus; merus 4.00–6.40 (median 4.71, n = 7) times as long as wide, 0.82–1.06 (median 0.94, n = 4) times as long as ischium. Third pereiopod (Fig. 6C, D) not sexually dimorphic, dactylus 4.00–4.67 (median 4.13, n = 4) times as long as wide (terminal claw and spiniform setae on flexor margin included), terminating in one large claw with 3–6 stout spiniform setae on flexor margin; propodus slender, 8.40–12.00 (median 9.22, n = 4) times as long as wide, 2.63–3.24 (median 2.92, n = 4) times as long as dactylus; carpus bearing one strong and three or four small spiniform setae on posterior margin of outer surface, 4.63–5.13 (median 4.86, n = 3) times as long as wide, 0.73–0.81 (median 0.75, n = 4) times as long as propodus; merus slender, 5.84–6.70 (median 6.46, n = 3) times as long as wide, 1.97–2.05 (median 1.97, n = 4) times as long as carpus, bearing 3–5 strong spiniform setae on posterior margin of outer surface. Ischium with one spiniform seta. Fifth pereiopod (Fig. 6E, F) slender, dactylus 5.67 times as long as wide (terminal claw and serrate setae on flexor margin included), terminating in one large claw with 34–36 serrate setae on flexor margin; propodus slender, 10.89–15.43 (median 13.16, n = 2) times as long as wide, 3.18 times length of dactylus, carpus bearing one strong and three small spiniform setae on posterior margin of outer surface, 0.53 times as long as propodus; merus slender, 7.83 times as long as wide, 1.81 times length of carpus, bearing two strong spiniform setae on posterior margin of outer surface. Ischium without a strong spiniform seta.

***Pleopods*.** Appendix masculina (Fig. 6G) on male second pleopod stick-like, with long spiniform setae on inner and distal margin, few pappose setae on basal part, appendix interna reaching to ~ 0.8 of appendix masculina.

###### Colouration.

Body dark reddish or brown with tiny light bluish dots, well-defined white transversal bands on the first, third, fifth, and sixth abdominal segments (Fig. 3H).

###### Reproductive biology and larval development.

Ovigerous females with few eggs (35, n = 1). Size of eggs 0.77–0.81 × 0.44–0.49 mm (n = 3).

###### Etymology.

The Latin word *fuscus* refers to the species’ dark reddish or brown colouration (Fig. 3H).

###### Distribution.

*Caridina fusca* sp. nov. is endemic to Lake Poso. Specimens were found at two localities within the lake, in a small bay south of the town of Tentena at the east shore and in a bay at the west shore.

###### Ecology.

*Caridina fusca* sp. nov. is found under rocks in deep water (more than 5 m depth), while the morphologically similar species *C. sarasinorum* is usually found on various kinds of substrate like deposits of leaf litter, on wood or macrophytes (von Rintelen and Cai 2009).

###### Remarks.

In life colouration, *C. fusca* sp. nov. might be confused with *C. sarasinorum*, also endemic to Lake Poso. In the latter, the transversal bands on the abdomen are less defined and scraggy compared to the sharply defined straight bands in *C. fusca* sp. nov. In preserved condition *C. fusca* sp. nov. can be differentiated from *C. sarasinorum* by the rostrum reaching to the end of the antennular peduncle, the dorsal and ventral margin armed throughout almost to the tip vs. reaching to the distal margin of the scaphocerite or beyond, unarmed in anterior one-third to half of the dorsal margin in *C. sarasinorum*. Epipods are reduced on the third maxilliped and absent on all pereiopods of *C. fusca* sp. nov. vs. well-developed on the third maxilliped and first pereiopod, absent on second to fifth pereiopods in *C. sarasinorum*. The chelae of the first pair of pereiopods are not inflated, 2.29–2.73 times as long as wide, 1.17–1.34 times as long as the carpus in *C. fusca* sp. nov. vs. slightly inflated, 1.74–2.10 times as long as wide, 1.35–1.48 times as long as the carpus in *C. sarasinorum*. The carpi of the first pair of pereiopods are more slender (2.33–4.00 times as long as wide) and hardly excavated distally vs. more stout (1.75–2.22 times as long as wide) and slightly excavated distally in *C. sarasinorum*.

##### Caridina lilianae

Taxon classificationAnimaliaDecapodaAtyidae

Klotz, Wowor & K. von Rintelen sp. nov.

6CDC012E-891C-5100-9469-C74124FDCE46

http://zoobank.org/89F09DAB-32A3-4C99-82C7-5400C8C2632F

Figures 3C , 7 , 8

###### Material examined.

***Holotype***: ov. ♀ cl. 3.1 mm (MZB Cru 5035), Indonesia, Central Sulawesi, Lake Poso, E shore, S of Tentena, dredge in centre of bay, C. Lukhaup, T. von Rintelen, C. and F. Logemann leg., 17 Jun. 2011. **Paratypes**: 3 ov. ♀♀ cl. 2.8–2.9 mm (MZB Cru 5036), 2 ov. ♀♀ cl. 2.7 and 3.1 mm, 1 ♀ cl. 2.7 mm, 1 ♂ cl. 2.5 mm (ZMB 29807), same data as holotype; 3 ♀♀ cl. 1.7–2.6 mm, 4 ♂♂ cl. 1.9–2.2 mm (MZB Cru 5037), 1 ov. ♀ cl. 2.4 mm, 2 ♀♀ cl. 1.9 and 2.5 mm, 3 ♂♂ cl. 1.9–2.2 mm (ZMB 30197), Lake Poso, E shore, small bay within mouth of outlet, 1°46.30'S, 120°38.38'E, W. Klotz and T. von Rintelen leg., 12 May 2017; 1 ov. ♀ cl. 2.6 mm, 2 ♀♀ cl. 2.1 and 2,6 mm, 2 ♂♂ cl. 1.8 and 2.4 mm (MZB 5038), 2 ov. ♀♀ cl. 2.5 and 2.7 mm, 2 ♀♀ cl. 2.6 mm, 3 ♂♂ cl. 2.0–3.0 mm (ZMB 30713), Lake Poso, W shore, Bay N of Bancea, in 1.5–3 m depth, 1°58.91'S, 120°34.877'E, T. von Rintelen leg., 04 Aug. 2018; 1 ov. ♀ cl. 2.4 mm, 1 ♀ cl. 2.3 mm (ZMB 30755), 2 ♀♀ cl. 2.1 and 2.3 mm, 1 ♂ cl. 1.9 mm (MZB Cru 5091) Lake Poso: E shore, small bay within mouth of outlet, 1°46.30'S, 120°38.38'E, T. von Rintelen leg., 14 Jul 2019.

###### Description.

**Figure 7.**
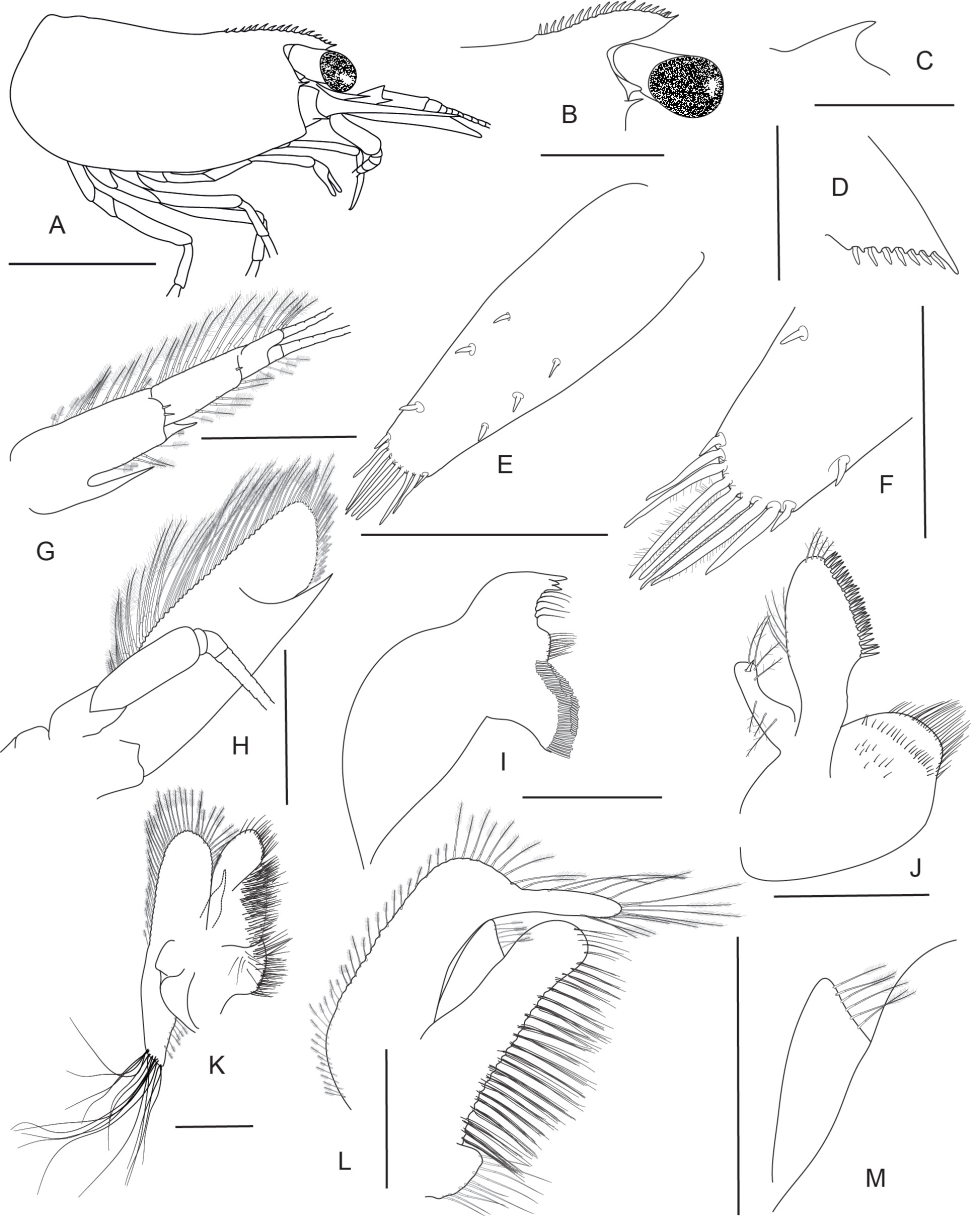
*Caridina lilianae* sp. nov. Morphology 1. Paratype ov. ♀, cl. 3.1 mm, ZMB 29807 **A** cephalothorax and cephalic appendages **C** preanal carina **D** uropodal diaeresis **G** antennular peduncle **H** scaphocerite **I** mandible **K** maxilla **L** first maxilliped **M** Palp of first maxilliped; paratype ♂, cl. 2.2 mm, ZMB 30197 **B** rostrum **E, F** telson **J** maxillula. Scale bars: 2 mm (**A**); 1 mm (**B, E**); 0.5 mm (**C, D, F, M**).

***Cephalothorax and cephalic appendages*.** Postorbital carapace length 1.7–3.1 mm (n = 33). Rostrum (Fig. 7A, B) very short, not overreaching distal margin of eyes, clearly convex on dorsal margin, abruptly tapering to a fine tip distally, 0.18–0.33 (median 0.26, n = 20) times as long as carapace, rostral formula 5–10 + 5–10 / 0. Antennal spine well separated from inferior orbital angle. Pterygostomial angle subrectangular. Eyes well developed with globular cornea. Antennular peduncle (Fig. 7A, G), 0.70–0.79 (median 0.75, n = 6) times as long as carapace in females, 0.92 (n = 1) times as long as carapace in male, first segment 2.08–2.70 (median 2.42, n = 7) times as long as second segment, second segment 1.67–2.75 (median 2.50, n = 7) times longer than third segment. Stylocerite reaching to 0.68–0.89 (median 0.77, n = 6) of first segment of antennular peduncle. Scaphocerite (Fig. 7H) 3.43–4.62 (median 4.02) times as long as wide.

***Abdominal somites, telson and uropods*.** Sixth abdominal somite 0.68–0.88 (median 0.77, n = 6) times carapace length, 1.78–2.26 (median 2.00, n = 6) times as long as fifth somite, 1.08–1.29 (median 1.22, n = 6) times as long as telson. Telson (Fig. 7E, F) with distal margin rounded or convex without a median projection, with 2–4 pairs of short spiniform setae dorsally and one pair of short spiniform setae dorsolaterally; distal end with 4–8 long spiniform setae, lateral pair shorter than others. Preanal carina (Fig. 7C) with a distinct hook-like spine. Uropodal diaeresis (Fig. 7D) with seven or eight stout movable spiniform setae, outermost ones shorter than lateral angle.

**Figure 8.**
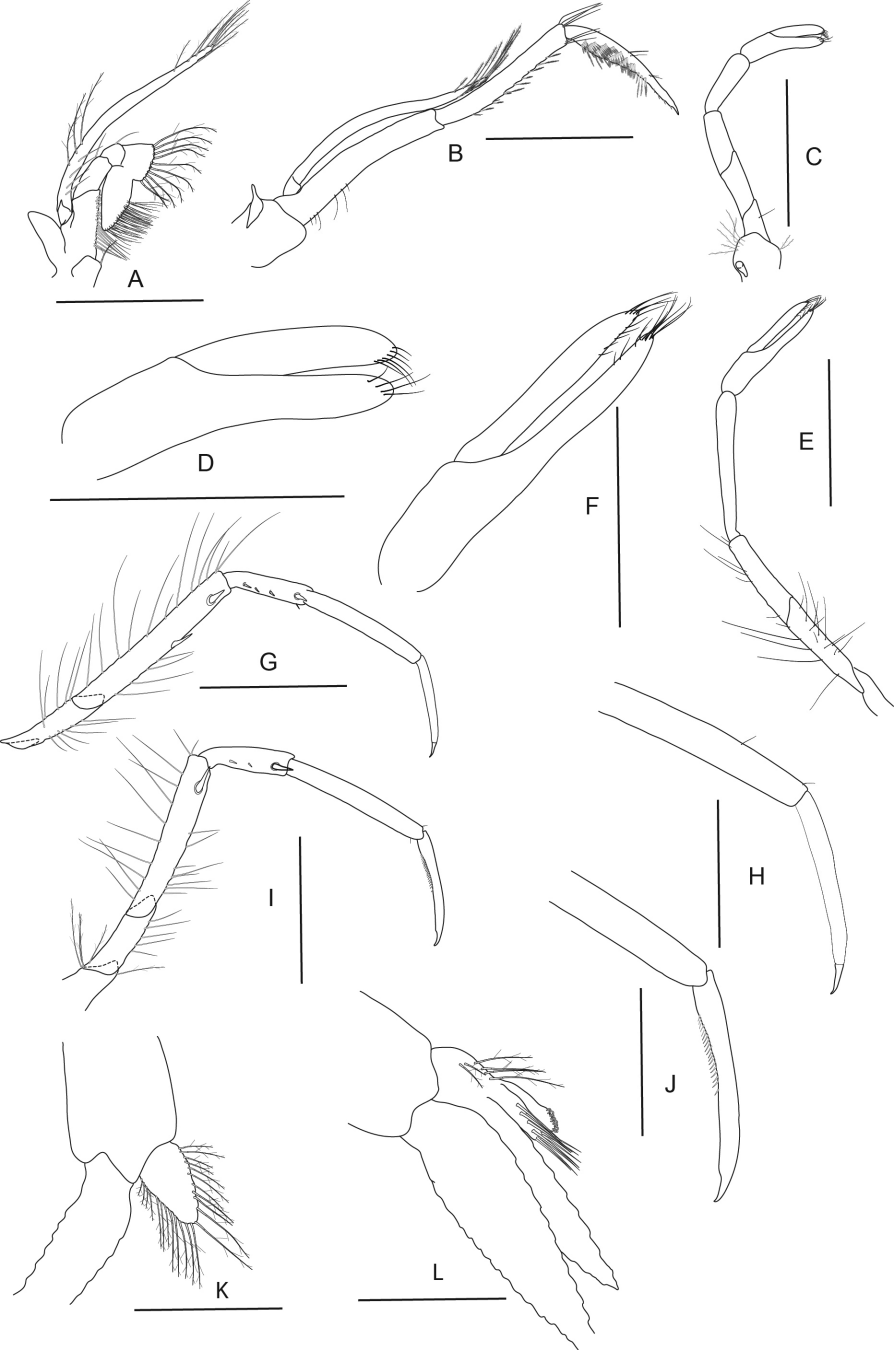
*Caridina lilianae* sp. nov. Morphology 2. Paratype ov. ♀, cl. 3.1 mm, ZMB 29807 **A** second maxilliped **B** third maxilliped **C** first pereiopod **D** chela of first pereiopod **E** second pereiopod **F** chela of second pereiopod **G** third pereiopod **H** dactylus of third pereiopod **I** fifth pereiopod **J** dactylus of fifth pereiopod; paratype ♂, cl. 2.2 mm, ZMB 30197 **K** endopod of male first pleopod **L** appendix masculina on male second pleopod. Scale bars: 1 mm (**A–C, E, G, I**); 0.5 mm (**D, F, H, J–L**).

***Mouthparts and branchiae*.** Incisor process of mandible (Fig. 7I) ending in irregular teeth, molar process truncated. Lower lacinia of maxillula (Fig. 7J) ovate, upper lacinia elongate, with numerous distinct teeth on inner margin, palp slender with few pappose setae and one conical spiniform seta near tip. Upper endites of maxilla (Fig. 7K) subdivided, palp slender, scaphognathite tapering posteriorly, fringed with long, curved setae at posterior margin. Palp of first maxilliped (Fig. 7L, M) ending in blunt triangular shape. Podobranch on second maxilliped (Fig. 8A) reduced to a lamina. Third maxilliped (Fig. 8B) with one well developed and one strongly reduced arthrobranch, ultimate segment slightly shorter than penultimate segment. First pereiopod with an arthrobranch. Pleurobranchs present on all pereiopods. Epipod reduced (without distal hook) on third maxilliped, absent from all pereiopods (a vestigial epipod was seen in one of the specimens examined (Fig. 8C)).

***Pereiopods*.** Chelae of first and second pereiopods (Fig. 8C–F) rather less developed and conspicuous small; chela of first pereiopod 3.43–4.62 (median 4.02, n = 2) times as long as wide, 0.94–0.96 (median 0.95, n = 2) times length of carpus; tips of fingers rounded, without hooks, with scarce hairs near tip; dactylus 1.50–1.58 (median 1.54, n = 2) times as long as palm; carpus slender, hardly excavated distally, 4.55–5.33 (median 4.94, n = 2) times as long as wide, 1.32–1.33 (median 1.32, n = 2) times length of merus. Merus 3.80–4.00 (median 3.90, n = 2) times as long as wide, as long as ischium, with few stiff simple setae. Chela of second pereiopod 4.31–4.92 (median 4.62, n = 2) times as long as wide, 0.80–0.90 (median 0.85, n = 2) times length of carpus; tips of fingers rounded, without hooks, with scarce hairs near tip; dactylus 1.67–1.80 (median 1.73, n = 2) times as long as palm; carpus 6.20–8.00 (median 7.10, n = 2) times as long as wide, 1.35–1.48 (median 1.41, n = 2) times as long as merus; merus 4.60–4.91 (median 4.75, n = 2) times as long as wide, as long as ischium, merus and ischium with long simple setae. Third pereiopod (Fig. 8G, H) slender, not sexually dimorphic, dactylus very slender 8.00–10.80 (median 9.40, n = 2) times as long as wide (terminal claw included), terminating in one large claw, without spiniform setae on flexor margin; propodus 7.25–7.56 (median 7.40, n = 2) times as long as wide, 1.26–1.45 (median 1.35, n = 2) times as long as dactylus; carpus 4.22–4.55 (median 4.38, n = 2) times as long as wide, 0.66–0.74 (median 0.70, n = 2) times as long as propodus; merus 6.33–7.43 (median 6.88, n = 2) times as long as wide, 1.31–1.53 (median 1.42, n = 2) times as long as carpus, bearing two strong spiniform setae on posterior margin of outer surface and long stiff simply setae along the entire segment. Ischium without spiniform seta but with long stiff simply setae similar to the setae on merus. Fifth pereiopod (Fig. 8I, J) slender, dactylus 10.00 times as long as wide (terminal claw and serrate setae on flexor margin included), terminating in one large claw with ~ 19 serrate setae on proximal half of flexor margin; propodus 8.00 times as long as wide, 1.33 times length of dactylus, carpus 4.17 times as long as wide, 0.63 times as long as propodus; merus 6.57 times as long as wide, 1.84 times length of carpus, bearing one strong spiniform seta on posterior margin of outer surface and long stiff simply setae along the entire segment. Ischium without spiniform seta but with long stiff simply setae similar to the setae on merus.

***Pleopods*.** Endopod of male first pleopod (Fig. 8K) subtriangular, without appendix interna, 2.00–2.22 (n = 2) times as long as proximal width. Appendix masculina on male second pleopod (Fig. 8L) slender, 7.60–10.33 (n = 2) times as long as wide, with long spiniform setae on inner and distal margin, few pappose setae on basal part, appendix interna reaching to distal margin of appendix masculina or slightly overreaching it.

###### Colouration.

Body colouration transparent to whitish with minute sand-coloured dots (Fig. 3C).

###### Reproductive biology and larval development.

Ovigerous females with few eggs (35, n = 1). Size of undeveloped eggs (early stage embryos without eyespot) 0.61–0.72 × 0.37–0.39 mm, size of developed eggs (late stage embryos with eyes) 0.70–0.76 × 0.39–0.44 mm (n = 6).

###### Etymology.

Named after the second and last authors’ first daughter who is very interested in field work and helped to observe and document this species while visiting the lake in 2019.

###### Distribution.

*Caridina lilianae* sp. nov. is endemic to Lake Poso. Specimens were found at three localities within the lake, two within a bay south of the town of Tentena at the east shore and one in a bay at the west shore.

###### Ecology.

*Caridina lilianae* sp. nov. lives on very fine sand or silt (soft substrate) in shallow water (1.5–2.5m).

###### Remarks.

With its small size and the less developed chelae with scarce setae at the tip of the fingers, *C. lilianae* sp. nov. is similar to *C. mayamareenae* sp. nov. but can easily be distinguished from this species by the very short, convex rostrum (vs. rostrum conspicuous high, reaching to end of second segment of antennular peduncle or slightly overreaching this segment) and the slender third pair of pereiopods bearing long stiff setae on merus and ischium but without any spiniform setae on flexor margin (vs. third pereiopod very robust, without long simple setae on merus and ischium and dactylus with five or six spiniform setae on flexor margin). These characters also distinguish *C. lilianae* sp. nov. from all other *Caridina* spp. known from the Lake Poso. Although *C. lilianae* sp. nov. and *C. mayamareenae* sp. nov. occur in sympatry in the lake, the microhabitats of these species are quite different. *Caridina mayamareenae* sp. nov. lives in empty shells of aquatic snails while *C. lilianae* sp. nov. on soft substrate. The long stiff simple setae attached to the posterior segments of the chelipeds and pereiopods could be interpreted as a morphological adaption to this kind of habitat by preventing them to subside into the soft substrate. This hypothesis would need to be tested, though. In the field, the whitish or cream-coloured body colouration is indiscernible on light-coloured sandy habitats (Fig. 3D).

##### Caridina marlenae

Taxon classificationAnimaliaDecapodaAtyidae

Klotz, Wowor & K. von Rintelen sp. nov.

2A9F9EBD-6099-58BC-B9A4-CB1614FA4B24

http://zoobank.org/801EC24A-93F5-48EF-9394-AF0930D50E36

Figures 3G , 9 , 10

###### Material examined.

***Holotype***: ♀ cl. 2.8 mm (MZB Cru 5039), Indonesia, Central Sulawesi, Lake Poso, E shore, S of Tentena, dive at small cape, 15 m, 1°46.394'S, 120°38.327'E, T. von Rintelen and W. Klotz leg., 12 May 2017. ***Paratypes***: 4 ♀♀ cl. 1.4–2.1 mm, 1 ♂ cl. 1.6 mm (MZB Cru 5040), 1 ♀ cl. 2.3 mm, 3 ♂♂ cl. 2.0–2.2 mm (ZMB 30199), same data as holotype; 2 ov. ♀♀ cl. 2.7 and 2.8 mm, 2 ♂♂ cl. 2.5 and 2.8 mm (MZB Cru 5041), 2 ♀♀ cl. 2.6 and 3.1 mm, 1 ♂ cl. 2.4 mm, 2 sequenced specimens without anterior pleopods cl. 2.2 and 2.9 mm (ZMB 29519), Lake Poso, E shore, S of Tentena, dive at small cape, in 15 m depth, 1°46.394'S, 120°38.327'E, M. Glaubrecht and T. von Rintelen leg., 16 May 2007; 2 ♀♀ cl. 2.1 and 2.5 mm, 1 ♂ cl. 2.0 mm, 2 juv. specimens (MZB Cru 5092), 2 ♀♀ cl. 1.9 and 2.2 mm, 1 ♂ cl. 1.8 mm, 1 juv. specimen (ZMB 31661), Lake Poso, E shore, S of Tentena, dive at small cape, in 15 m depth, 1°46.394'S, 120°38.327'E, T. von Rintelen leg., 14 Jul 2019.

###### Comparative material examined.

*Caridina sarasinorum* Schenkel, 1902, 1 ov. ♀ cl. 3.0 mm, 1 ♂ cl. 2.6 mm (ZMB 29288), Lake Poso, E shore, 2°0.825'S, 120°42.007'E, K. Zitzler leg., 16 Aug. 2004; 2 ov. ♀♀ cl. 3.2 and 3.6 mm, 1 ♀ cl. 3.mm, 5 ♂♂ cl. 2.1–2.6 mm, 2 juv. specimens cl. 1.7 mm, 1 damaged specimen cl. 2.0 mm (ZMB 30224), Lake Poso, E shore, beach in front of Dolidi Ndano Cottages, 1°48.14'S, 120°38.043'E, W. Klotz leg., 12 May 2017.

###### Description.

**Figure 9.**
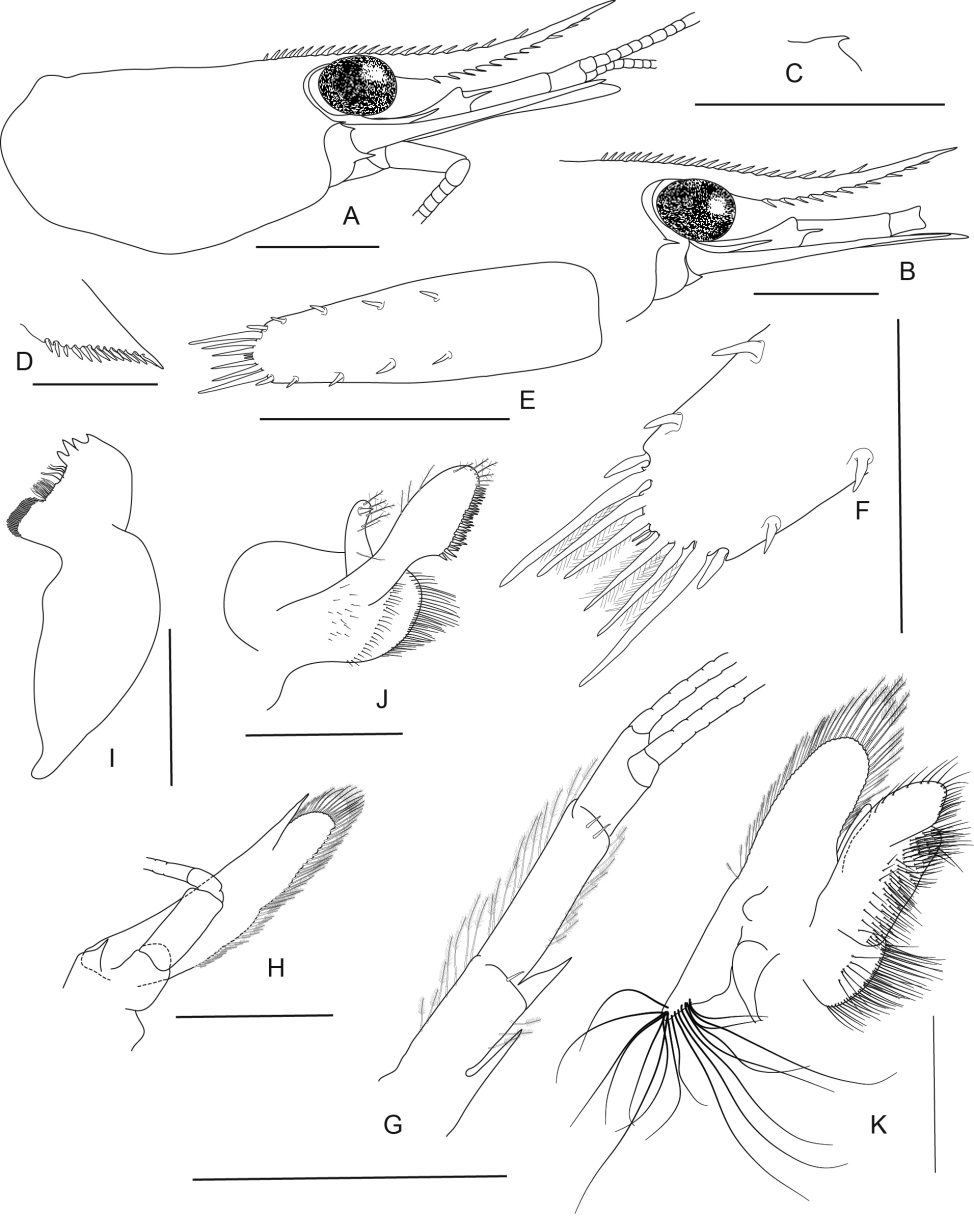
*Caridina marlenae* sp. nov. Morphology 1. Paratype ♂, cl. 2.2 mm, ZMB 30199 **A** cephalothorax and cephalic appendages **B** rostrum **C** preanal carina **D** uropodal diaeresis **E, F** telson **G** antennular peduncle **H** scaphocerite **I** mandible **K** maxilla; paratype ♀, cl. 2.3 mm, ZMB 30199 **J** maxillula. Scale bars: 1 mm (**A–E**); 0.5 mm (**F–K**).

**Cephalothorax and cephalic appendages.** Postorbital carapace length 1.44–3.07 mm (n = 19). Rostrum (Fig. 9A, B) long and slender, curved upwards or sigmoid, reaching slightly beyond end of scaphocerite or in small specimen, to end of the antennular peduncle, distal 0.16–0.40 (median 0.38, n = 11) of dorsal margin unarmed, ventral margin armed throughout, dorsal teeth more widely spaced distally, 0.95–1.54 (median 1.20, n = 14) times as long as carapace, rostral formula 3–6 (4–6) + 10–20 / 6–18. Antennal spine slightly separated from orbital margin. Pterygostomial angle broadly rounded. Eyes well developed with globular cornea. Antennular peduncle (Fig. 9A, B, G), 0.89–1.04 (median 0.96, n = 5) times as long as carapace, first segment 1.56–1.79 (median 1.71, n = 4) times as long as second segment, second segment 2.40–2.80 (median 2.58, n = 4) times longer than third segment. Tooth on distolateral margin of first segment of antennular peduncle acute. Stylocerite reaching to 0.78–0.83 (median 0.80, n = 4) of first segment of antennular peduncle. Scaphocerite (Fig. 9H) 4.10–4.25 (median 4.20, n = 3) times as long as wide.

***Abdominal somites, telson and uropods*.** Sixth abdominal somite 0.55–0.64 (median 0.62, n = 5) times carapace length, 1.93–2.23 (median 2.15, n = 5) times as long as fifth somite, 1.00–1.13 (median 1.12, n = 4) times as long as telson. Distal margin of telson (Fig. 9E, F) convex or subtriangular without a median projection, with 3–5 pairs of short spiniform setae dorsally and one pair of short spiniform setae dorsolaterally; distal end with 7–10 long spiniform setae, lateral pair slightly longer than others, innermost pair very tiny. Preanal carina (Fig. 9C) with a hook-like spine. Uropodal diaeresis (Fig. 9D) with 11–14 stout movable spiniform setae, outermost ones shorter than lateral angle.

**Figure 10.**
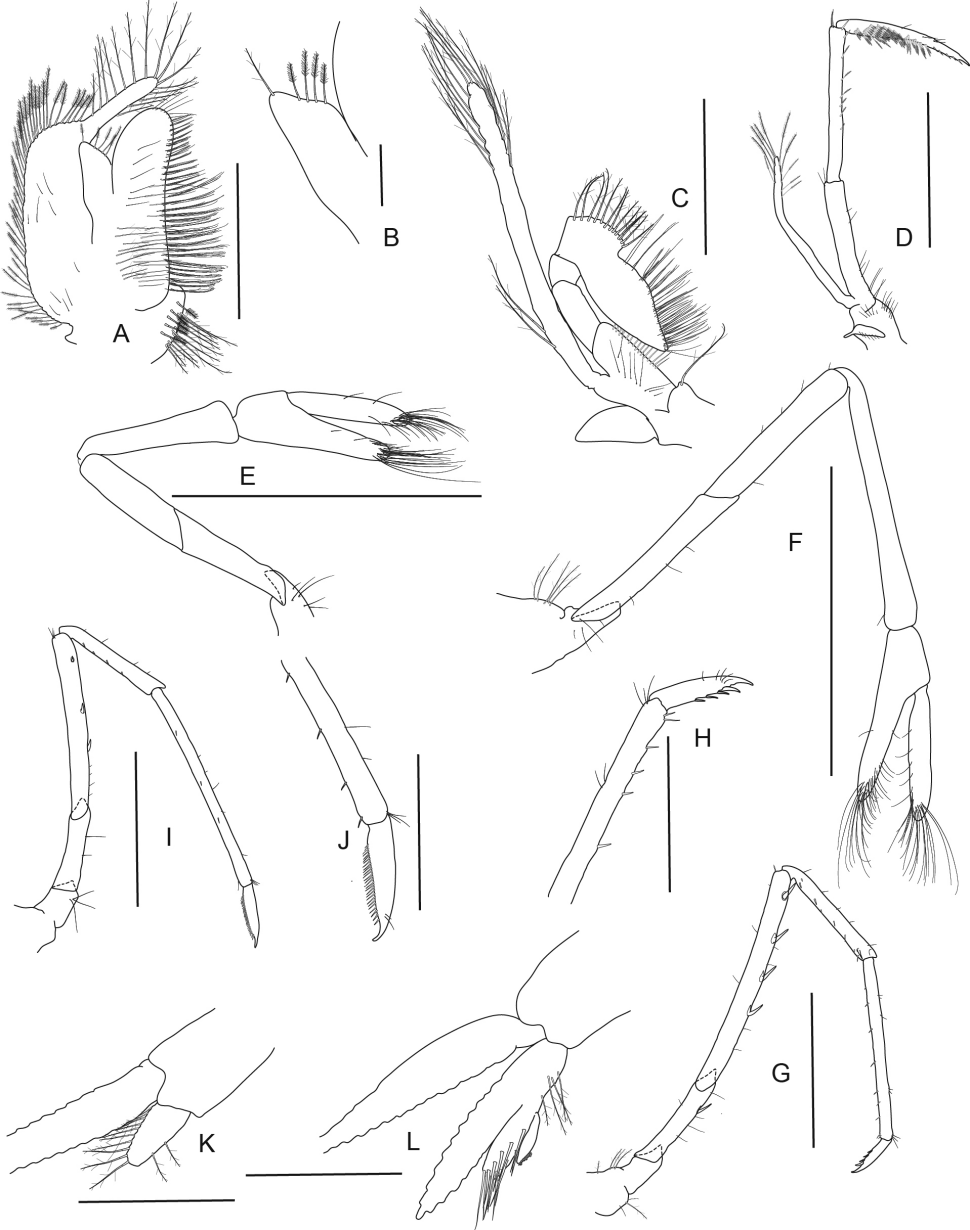
*Caridina marlenae* sp. nov. Morphology 2. Paratype ♂, cl. 2.2 mm, ZMB 30199 **A** first maxilliped **B** palp of first maxilliped **C** second maxilliped **D** third maxilliped **E** first pereiopod **F** second pereiopod **I** fifth pereiopod **K** endopod of male first pleopod **L** appendix masculina on male second pleopod; paratype ♀, cl. 2.3 mm, ZMB 30199 **G** third pereiopod **H** dactylus of third pereiopod **J** dactylus of fifth pereiopod. Scale bars: 0.5 mm (**A, C, H, J–L**); 0.1 mm (**B**); 1 mm (**D–G, I**).

***Mouthparts and branchiae*.** Incisor process of mandible (Fig. 9I) ending in irregular teeth, molar process truncated. Lower lacinia of maxillula (Fig. 9J) broadly rounded, upper lacinia elongate, with numerous distinct teeth on inner margin, palp slender with few pappose setae and one conical spiniform seta near tip. Upper endites of maxilla (Fig. 9K) subdivided, palp slender, scaphognathite tapering posteriorly, fringed with long, curved setae at posterior margin. Palp of first maxilliped (Fig. 10A, B) ending in triangular shape. Podobranch on second maxilliped (Fig. 10C) reduced to a lamina. Third maxilliped (Fig. 10D) with one well developed and one small arthrobranch, ultimate segment of maxilliped shorter than penultimate segment. First pereiopod with an arthrobranch. Pleurobranchs present on all pereiopods. Epipod slightly reduced (without distal hook) on third maxilliped, absent from all pereiopods.

***Pereiopods*.** Chelae of first and second pereiopod (Fig. 10E, F) well developed; chela of first pereiopod 3.00–3.83 (median 3.20, n = 5) times as long as wide, 1.00–1.05 (median 1.03, n = 5) times length of carpus; tips of fingers (Fig. 10E) rounded, without hooks, with tufts of hairs near tip; dactylus 1.88–2.50 (median 2.00, n = 5) times as long as palm; carpus slender, hardly excavated distally, 3.85–4.77 (median 4.00, n = 5) times as long as wide, 1.25–1.33 (median 1.29, n = 5) times length of merus. Merus 3.80–4.25 (median 4.00, n = 5) times as long as wide, 0.89–0.95 (median 0.92, n = 4) times as long as ischium. Chela of second pereiopod 3.69–4.38 (median 3.71, n = 5) times as long as wide, 0.68–0.79 (median 0.71, n = 6) times length of carpus; tips of fingers rounded, without hooks, with tufts of hairs near tip; dactylus 1.80–2.25 (median 2.00, n = 5) times as long as palm; carpus 6.78–9.80 (median 7.60, n = 5) times as long as wide, 1.27–1.58 (median 1.47, n = 4) times as long as merus; merus 5.33–6.50 (median 6.00, n = 4) times as long as wide, 0.82–0.93 (median 0.86, n = 4) times as long as ischium. Third pereiopod (Fig. 10G, H) not sexually dimorphic, dactylus 4.40–5.33 (median 4.50, n = 5) times as long as wide (terminal claw and spiniform setae on flexor margin included), terminating in one large claw with four or five short spiniform setae on flexor margin; propodus slender, 13.67–16.25 (median 14.57, n = 5) times as long as wide, 3.56–4.06 (median 3.78, n = 5) times as long as dactylus; carpus bearing one strong and 6 small short spiniform setae on posterior margin of outer surface, 7.50–8.36 (median 8.00, n = 5) times as long as wide, 0.63–0.75 (median 0.68, n = 5) times as long as propodus; merus slender, 10.36–12.62 (median 11.60, n = 5) times as long as wide, 1.68–1.93 (median 1.78, n = 5) times as long as carpus, bearing 3–5 strong spiniform setae on posterior margin of outer surface. Ischium with one spiniform seta. Fifth pereiopod (Fig. 10I, J) slender, dactylus 3.50–4.86 (median 4.13, n = 4) times as long as wide (terminal claw and serrate setae on flexor margin included), terminating in one large claw with 24–31 serrate setae on flexor margin; propodus slender, 13.43–16.00 (median 14.00, n = 4) times as long as wide, 3.29–3.50 (median 3.33, n = 4) times length of dactylus, carpus bearing one strong and 5–7 small spiniform setae on posterior margin of outer surface, 7.57–9.00 (median 7.88, n = 4) times as long as wide, 0.55–0.64 (median 0.57, n = 4) times as long as propodus, 0.63–0.70 (median 0.66, n = 4) times as long as merus; merus slender, 9.20–10.67 (median 10.55, n = 4) times as long as wide, 1.44–1.53 (median 1.51, n = 4) times length of carpus, bearing three or four strong spiniform setae on posterior margin of outer surface. Ischium without a strong spiniform seta.

***Pleopods*.** Endopod of male first pleopod (Fig. 10K) subtriangular, without an appendix interna, two pappose setae on outer, ~ eight on inner margin, 1.91–2.57 (median 2.00, n = 3) times as long as proximal width, 0.29–0.41 (median 0.29, n = 3) times as long as exopod. Appendix masculina on male second pleopod (Fig. 10L) rod-shaped, 6.00–8.40 (median 6.00, n = 3) times as long as wide, with long spiniform setae on inner and distal margin, few pappose setae on basal part, appendix interna reaching to ~ 0.72–0.80 (median 0.80, n = 3) of appendix masculina.

###### Colouration.

Body colouration bright reddish with large white dots (Fig. 3G).

###### Reproductive biology and larval development.

Ovigerous females with few eggs (9, n = 1). Size of eggs 0.81–0.83 × 0.48–0.0.51 mm (n = 2).

###### Etymology.

Named after the second and last authors’ second daughter who is very interested in field work and helped to observe and document this species while visiting the lake in 2019.

###### Distribution.

*Caridina marlenae* sp. nov. is endemic to Lake Poso. Specimens were found only at one locality in a bay south of the town of Tentena at the east shore of the lake.

###### Ecology.

*Caridina marlenae* sp. nov. is found under rocks in deep water (more than 5 m).

###### Remarks.

With its long rostrum, approximately anterior 0.4 unarmed, *C. marlenae* sp. nov. is similar to *C. sarasinorum*, *C. schenkeli* and *C. longidigita*, all endemic to Lake Poso. In the field, body colouration alone is sufficient to differ *C. marlenae* sp. nov. from *C. sarasinorum* or *C. schenkeli* but it might be confused with reddish specimens of *C. longidigita*. *Caridina marlenae* sp. nov. is showing large bright white dots on reddish colouration of the entire body. In *C. sarasinorum*, the body is coloured dark brown with faint light transversal bands on first, third, fifth and sixth abdominal segments (W. Klotz, pers. observation on the comparative material listed above). In *C. schenkeli* the colouration of the body is mostly transparent with some brownish or whitish blotches.

In preserved condition, *C. marlenae* sp. nov. can be distinguished from *C. sarasinorum* by the more reduced epipods (slightly reduced on the third maxilliped, absent from all pereiopods vs. well developed (with distal hooks) on the third maxilliped and first pereiopod in *C. sarasinorum* and the slender chelipeds and pereiopods (in detail: chela of first pereiopod 3.00–3.83 times as long as wide vs. 1.74–2.1 times in *C. sarasinorum*, 1.00–1.05 times as long as carpus vs. 1.35–1.48 times in *C. sarasinorum*, dactylus 1.88–2.50 times as long as palm vs. 0.83–1.05 times as long in *C. sarasinorum*, carpus 3.85–4.77 times as long as wide vs. 1.75–2.22 times in *C. sarasinorum*, merus 3.80–4.25 times as long as wide vs. 1.78–2.63 times in *C. sarasinorum*. Chela of second pereiopod 3.69–4.38 times as long as wide vs. 2.19–2.64 times in *C. sarasinorum*, dactylus 1.80–2.25 times as long as palm vs. 1.05–1.33 times as long in *C. sarasinorum*, carpus 6.78–9.80 times as long as wide vs. 4.56–5.05 times in *C. sarasinorum*, merus 5.33–6.50 times as long as wide vs. 3.60–4.29 times in *C. sarasinorum*. Dactylus of third pereiopod 4.40–5.33 times as long as wide vs. 4.0–4.20 in *C. sarasinorum*, propodus 13.67–16.25 times as long as wide vs. 10.00–11.11 in *C. sarasinorum*, 3.56–4.06 times as long as dactylus vs. 2.86–3.13 times as long as dactylus in *C. sarasinorum*. Propodus of fifth pereiopod 13.43–16.00 times as long as wide vs. 10.00–12.00 in *C. sarasinorum*).

*Caridina marlenae* sp. nov. can be distinguished from *C. schenkeli* by the more reduced epipods (slightly reduced on the third maxilliped, absent from all pereiopods vs. well developed (with distal hooks) on the third maxilliped and first and second pereiopod in *C. schenkeli* and the slender chelipeds and armature of the dactyli of pereiopods (in detail: chela of first pereiopod 3.00–3.83 times as long as wide vs. 1.90–3.2 times in *C. schenkeli*, dactylus 1.88–2.50 times as long as palm vs. 1.0–1.4 times as long in *C. schenkeli*. Carpus of first cheliped 3.85–4.77 times as long as wide vs. 2.1–3.2 times as long as wide in *C. schenkeli*. Dactylus of second pereiopod 1.80–2.25 times as long as palm vs. 1.2–1.4 times as long in *C. schenkeli*, carpus 6.78–9.80 times as long as wide vs. 4.5–6.5 times in *C. schenkeli*. Dactylus of third pereiopod with four or five spiniform setae on flexor margin vs. with 6–8 spiniform setae in *C. schenkeli*. Dactylus of fifth pereiopod with 24–31 serrate setae on flexor margin vs. with 57–64 in *C. schenkeli*.

*Caridina marlenae* sp. nov. can also be distinguished from *C. longidigita* by the type of chelae built for scraping vs. for filter-feeding in *C. longidigita* (brushes of setae short on tips of fingers of chelipeds vs. setae long, chela of first cheliped 3.00–3.83 times as long as wide vs. 4.6–6.5 times as long as wide in *C. longidigita*, dactylus 1.88–2.50 times as long as palm vs. 3.6–4.6 times as long in *C. longidigita*, carpus 3.85–4.77 times as long as wide vs. 4.8–8.1 times in *C. longidigita*; chela of second pereiopod 3.69–4.38 times as long as wide vs. 4.8–6.4 times in *C. longidigita*, dactylus 1.80–2.25 times as long as palm vs. 3.4–3.9 times as long in *C. longidigita*).

##### Caridina mayamareenae

Taxon classificationAnimaliaDecapodaAtyidae

Klotz, Wowor & K. von Rintelen sp. nov.

6E649782-D755-5AC5-B68A-151C9A454832

http://zoobank.org/038EA514-2161-42BF-8F93-6F8CF696B917

**Figure 11.**
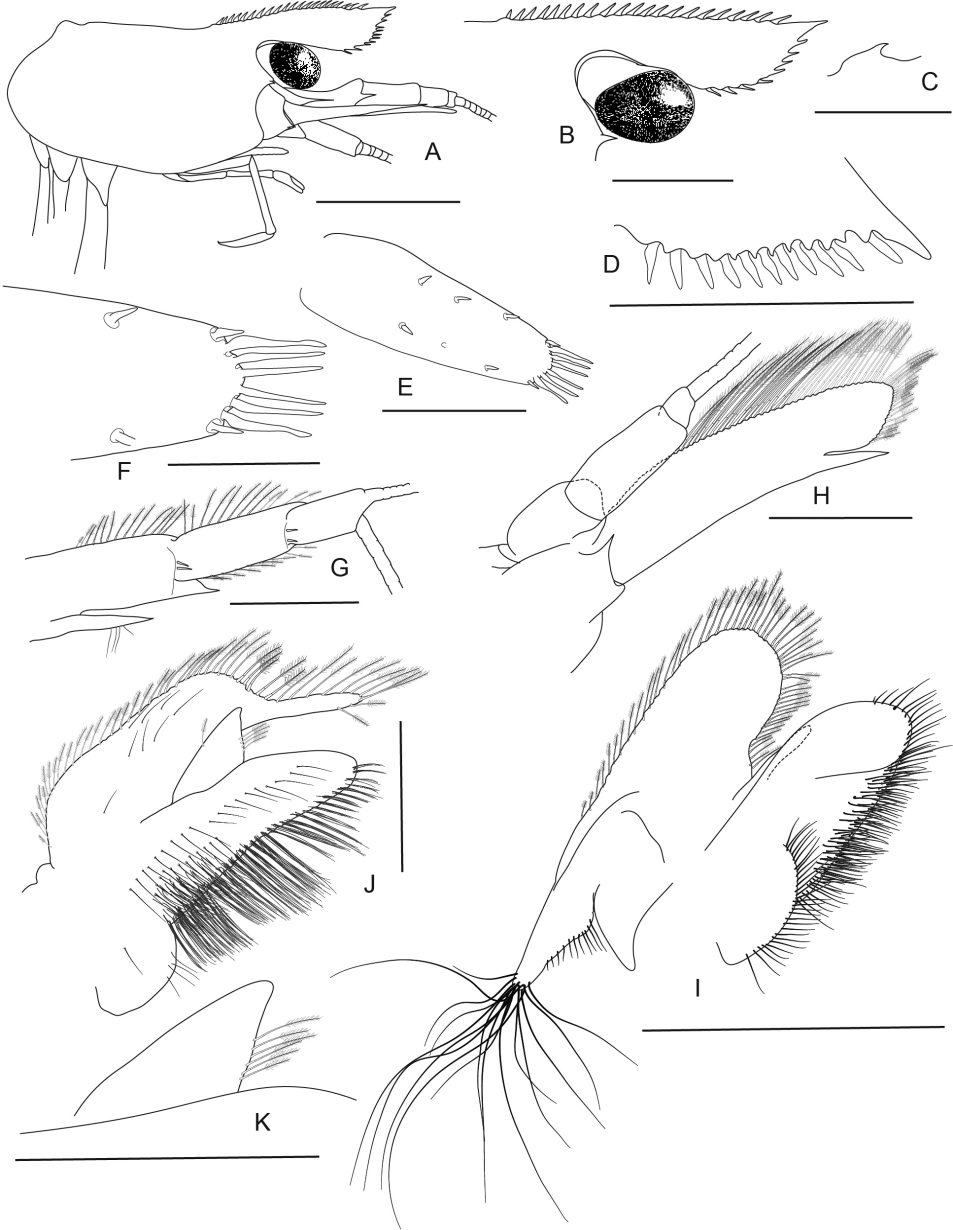
*Caridina mayamareenae* sp. nov. Morphology 1. Paratype ♂, cl. 2.7 mm, ZMB 29627 **A** cephalothorax and cephalic appendages **C** preanal carina **D** uropodal diaeresis **E–F** telson **G** antennular peduncle **H** scaphocerite **I** maxilla **J** first maxilliped **K** palp of first maxilliped; paratype ♀, cl. 2.7 mm, MZB Cru 5044 **B** rostrum. Scale bars: 2 mm (**A, E**); 1 mm (**B, G–I**); 0.5 mm (**C, D, F, J, K**).

**Figure 12.**
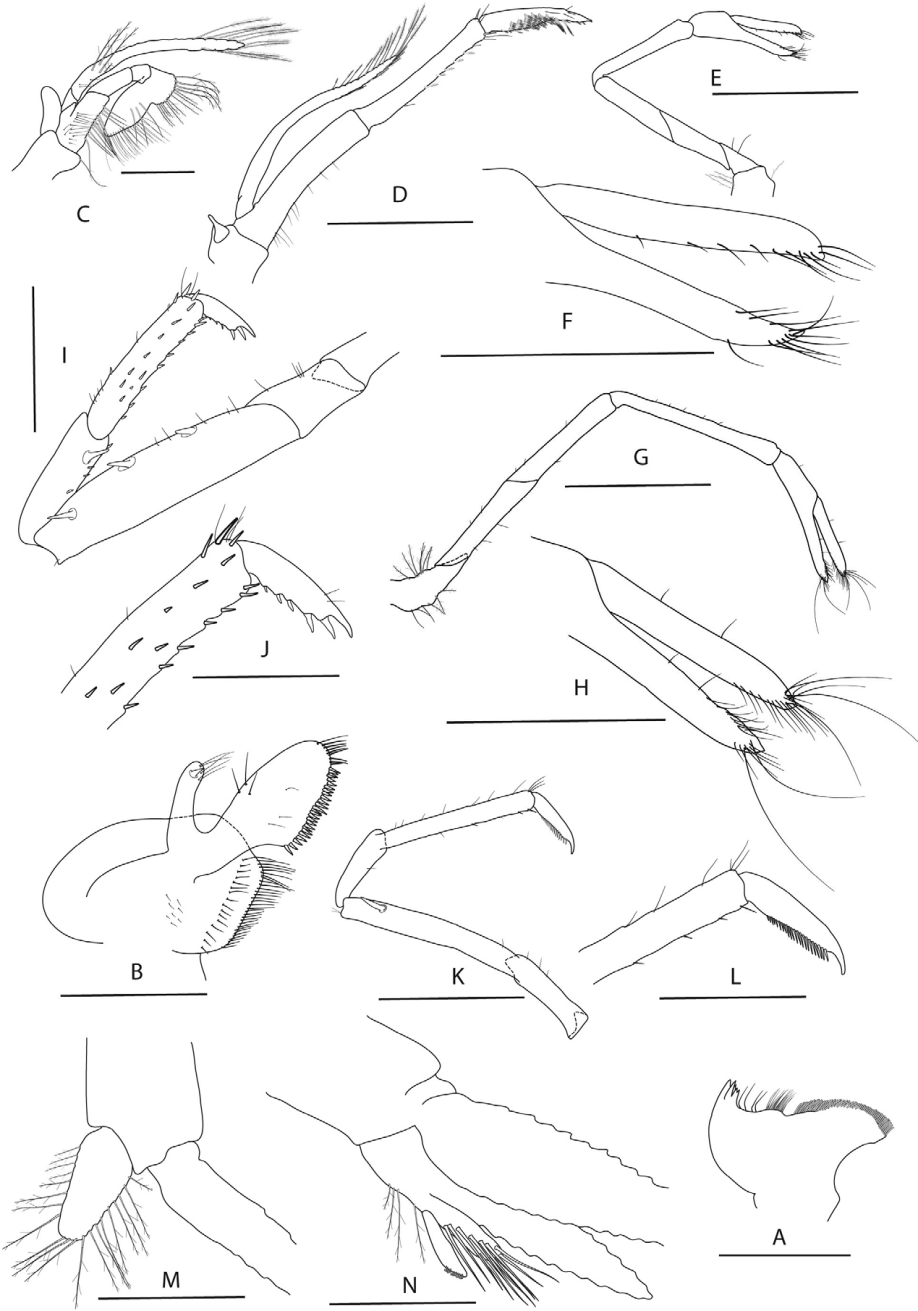
*Caridina mayamareenae* sp. nov. Morphology 2. Paratype ♂, cl. 2.7 mm, ZMB 29627 **A** distal part of mandible **B** maxillula **C** second maxilliped **D** third maxilliped **E** first pereiopod **F** dactyli of first pereiopod **I** third pereiopod **J** dactylus of third pereiopod **K** fifth pereiopod **L** dactylus of fifth pereiopod **M** endopod of male first pleopod **N** appendix masculina on male second pleopod; paratype ♀, cl. 2.7 mm, MZB Cru 5044 **G** second pereiopod **H** dactyli of second pereiopod. Scale bars: 0.5 mm (**A–C, F, H, J, L–N**); 1 mm (**D, E, G, I, K**).

Figures 2E , 3A–B , 11 , 12

###### Material examined.

***Holotype***: ov. ♀ cl. 3.0 mm (MZB Cru 5042), Indonesia, Central Sulawesi, Lake Poso, E shore, S of Tentena, dive at small cape, 15 m, 1°46.394'S, 120°38.327'E, J. Pfaender and T. von Rintelen leg., 21 Sep. 2015. ***Paratypes***: 2 ov. ♀♀ cl. 2.6 and 2.8 mm, 4 ♀♀ cl. 2.3–3.4 mm, 3 ♂♂ cl. 1.7–2.5 mm, 1 juv. cl. 1.5 mm (MZB Cru 5043), same data as holotype; 2 ♂♂ cl. 2.5 and 2.7 mm, 1 ov. ♀ cl. 2.8 mm, 1 incomplete ♀ cl. 2.7 mm (ZMB 29627), Lake Poso, E shore, S of Tentena, dredge in centre of bay, T. von Rintelen, C. Lukhaup and F. Logemann leg., 17 Jun. 2011; 1 ♀ cl. 2.7 mm (MZB Cru 5044), Lake Poso, E shore, “Sulawesi Rock”, 1°56.102'S, 120°40.402'E, J. Pfaender and T. von Rintelen leg., 22 Sep. 2015; 4 ♂♂ cl. 2.0–2.3 mm, 1 ov. ♀ cl. 2.9 mm (ZMB 29620), Lake Poso, E shore, beach in front of Dolidi Ndano Cottages, 1°48.14'S, 120°38.043'E, J. Pfaender and T. von Rintelen leg., 22 Sep. 2015; 30 ♂♂ cl. 1.5–2.7 mm, 5 ♀♀ cl. 1.6–2.9 mm, 4 ov. ♀♀ cl. 2.7–3.0 mm, 4 juv. cl. 1.4–1.7 mm (ZMB 30202), 34 ♂♂ cl. 1.5–2.6 mm, 6 ♀♀ cl. 2.0–3.0 mm, 2 ov. ♀♀ cl. 2.5 and 3.1 mm, 7 juv. cl. 1.3–1.6 mm (MZB Cru 5045) , Lake Poso, E shore, beach in front of Dolidi Ndano Cottages, dive to 15 m, 1°48.14'S, 120°38.043'E, T. von Rintelen leg., 09 May 2017; 4 ov. ♀♀ cl. 2.8–2.9 mm, 8 ♀♀ cl. 1.8–2.8 mm, 6 ♂♂ cl. 3.6–3.8 mm (ZMB 30709), 4 ov. ♀♀ cl. 2.7–3.0 mm, 5 ♂♂ cl. 1.8–2.2 mm, 8 ♀♀ cl. 1.3–2.3 mm (MZB Cru 5046), Lake Poso, NW shore, westernmost cape, 15 m depth, 1°47.39'S, 120°32.641'E, T. von Rintelen leg., 03 Aug. 2018; 5 ov. ♀♀ cl. 2.8–3.0 mm, 4 ♀♀ cl. 1.5–2.8 mm, 6 ♂♂ cl. 1.9–2.1 mm (ZMB 30710), 5 ov. ♀♀ cl. 2.6–2.7 mm, 3 ♀♀ cl. 1.5–2.7 mm, 7 ♂♂ cl. 1.5–2.0 mm (MZB Cru 5047), Lake Poso, NW shore, westernmost cape, 10 m depth, 1°47.39'S, 120°32.641'E, T. von Rintelen leg., 03 Aug. 2018; 2 ov. ♀♀ cl. 2.6 and 2.7 mm, 5 ♀♀ cl. 2.0–2.6 mm, 5 ♂♂ cl. 1.5–1.8 mm (ZMB 30711), 1 ov. ♀ cl. 2.6 mm, 5 ♀♀ cl. 1.6–2.2 mm, 5 ♂♂ cl. 1.5–2.5 mm (MZB Cru 5048), Lake Poso, W shore, S corner of Siuri bay, 13–14 m depth, 1°48.35'S, 120°31.703'E, T. von Rintelen leg., 03 Aug. 2018; 3 ov. ♀♀ cl. 2.5–2.7 mm, 4 ♀♀ cl. 1.8–3.0 mm (ZMB 30714), 4 ov. ♀♀ cl. 2.2–3.0 mm, 1 ♀ cl. 3.4 mm, 1 carapace sex unknown cl. 2.7 mm (MZB Cru 5049), Lake Poso, W shore, Bay N of Bancea, 15 m depth, 1°58.91'S, 120°34.877'E, T. von Rintelen leg., 04 Aug. 2018. 1 ov. ♀ cl. 2.7 mm (MZB Cru 5093), Lake Poso, E shore, small bay within mouth of outlet, 1°46.30'S, 120°38.38'E, T. von Rintelen leg., 14 Jul 2019. 1 ♀ cl. 2.5 mm (ZMB 30388), 1 ♂ cl. 1.8 mm (MZB Cru 5097), Lake Poso, E shore, “Sulawesi Rock”, 1°56.102'S, 120°40.402'E, T. von Rintelen leg., 21 Sep. 2019.

###### Description.

***Cephalothorax and cephalic appendages*.** Postorbital carapace length 1.3–3.8 mm (n = 220). Rostrum (Fig. 11A, B) conspicuously high, straight or slightly convex on dorsal margin, distal part of ventral margin convex, reaching to end of second segment of antennular peduncle or slightly overreaching this segment, ventral teeth placed at convex part close to the tip, 0.66–1.0 (median 0.81, n = 7) times as long as carapace, rostral formula 4–7 (5) + 10–19 (15) / 4–12 (7–9). Small antennal spine well separated from inferior orbital angle. Pterygostomial angle broadly rounded. Eyes well developed with globular cornea. Antennular peduncle (Fig. 11A, G), 0.72–0.83 (median 0.79, n = 4) times as long as carapace in females, 0.95–1.13 (median 1.09, n = 3) times as long as carapace in males, first segment 1.73–2.17 (median 2.0, n = 3) times as long as second segment, second segment 2.30–2.60 (median 2.50) times longer than third segment. Stylocerite reaching to 0.77–0.80 (median 0.77, n = 4) of first segment of antennular peduncle. Scaphocerite (Fig. 11H) 3.48–3.64 (median 3.56) times as long as wide.

***Abdominal somites, telson and uropods*.** Sixth abdominal somite 0.46–0.71 (median 0.58, n = 8) times carapace length, 1.65–2.0 (median 1.74, n = 8) times as long as fifth somite, 0.90–1.12 (median 1.04, n = 8) times as long as telson. Telson (Fig. 11E, F) 2.45–2.64 (n = 2) times as long as proximal wide, distal margin broadly convex without a median projection, with three or four pairs of short spiniform setae dorsally and one pair of short spiniform setae dorsolaterally; distal end with 6 long spiniform setae, lateral pair slightly longer than others. Preanal carina (Fig. 11C) with a hook-like spine. Uropodal diaeresis (Fig. 11D) with 11–13 stout movable spiniform setae, outermost ones shorter than lateral angle.

***Mouthparts and branchiae*.** Incisor process of mandible (Fig. 12A) ending in irregular teeth, molar process truncated. Lower lacinia of maxillula (Fig. 12B) broadly rounded, upper lacinia elongate, with numerous distinct teeth on inner margin, palp slender with few simple setae and one conical spiniform seta near tip. Upper endites of maxilla (Fig. 11I) subdivided, palp slender, scaphognathite tapering posteriorly, fringed with long, curved setae at posterior margin. End of palp of first maxilliped (Fig. 11J, K) ending in blunt triangular shape. Podobranch on second maxilliped (Fig. 12C) reduced to a lamina. Third maxilliped (Fig. 12D) with one well developed and one arthrobranch reduced to a small worm-like structure. First pereiopod with an arthrobranch. Pleurobranchs present on all pereiopods. Epipod vestigial on third maxilliped, absent from all pereiopods.

***Pereiopods*.** Chelae of first and second pereiopods (Fig. 12E–H) rather less developed and conspicuous small; chela of first pereiopod 4.00–4.17 (median 4.00, n = 3) times as long as wide, 1.07–1.14 (median 1.07, n = 3) times length of carpus; tips of fingers (Fig. 12E, F) rounded, without hooks, with scarce hairs near tip; dactylus 1.70–1.74 (median 1.73, n = 3) times as long as palm; carpus hardly excavated distally, 4.31–4.40 (median 4.31, n = 3) times as long as wide, 1.05–1.22 (median 1.22, n = 3) times length of merus. Merus 3.83–5.25 (median 4.18, n = 3) times as long as wide, as long as ischium. Chela of second pereiopod 5.00–5.27 (median 5.14, n = 3) times as long as wide, 0.73–0.81 (median 0.76, n = 3) times length of carpus; tips of fingers rounded, without hooks, with scarce hairs near tip; dactylus 1.47–1.93 (median 1.58, n = 3) times as long as palm; carpus 7.17–8.44 (median 8.17, n = 3) times as long as wide, 1.27–1.40 (median 1.34, n = 3) times as long as merus; merus 5.82–7.50 (median 6.36, n = 3) times as long as wide, as long as ischium. Third pereiopod (Fig. 12I, J) conspicuous stout, not sexually dimorphic, dactylus 3.40–4.38 (median 4.00, n = 3) times as long as wide (terminal claw and spiniform setae on flexor margin included), terminating in one large claw with five or six short spiniform setae on flexor margin; propodus stout, 5.25–7.80 (median 6.00, n = 3) times as long as wide, 2.10–2.47 (median 2.23, n = 3) times as long as dactylus; carpus 3.52–4.83 (median 3.94, n = 3) times as long as wide, 0.74–0.88 (median 0.85, n = 3) times as long as propodus; merus very stout, 4.67–6.50 (median 5.00, n = 3) times as long as wide, 1.89–2.24 (median 2.11, n = 3) times as long as carpus, bearing three strong spiniform setae on posterior margin of outer surface. Ischium without or with one spiniform seta. Fifth pereiopod (Fig. 12K, L) slender, dactylus 2.73–5.22 (median 5.00, n = 3) times as long as wide (terminal claw and serrate setae on flexor margin included), terminating in one large claw with 18–36 serrate setae on flexor margin; propodus 8.33–11.11 (median 9.09, n = 3) times as long as wide, 2.13–3.33 (median 2.50, n = 3) times length of dactylus, carpus 4.83–6.00 (median 5.17, n = 3) times as long as wide, 0.54–0.62 (median 0.58, n = 3) times as long as propodus; merus 6.93–10.00 (median 8.67, n = 3) times as long as wide, 1.68–1.85 (median 1.79, n = 3) times length of carpus, bearing 2–4 strong spiniform setae on posterior margin of outer surface. Ischium without a strong spiniform seta.

***Pleopods*.** Endopod of male first pleopod (Fig. 12M) subtriangular, without an appendix interna, 1.83 times as long as proximal width, 0.28 times as long as exopod. Appendix masculina on male second pleopod (Fig. 12N) slender, rod-shaped, 7.20 times as long as wide, with long spiniform setae on inner and distal margin, few pappose setae on basal part, appendix interna reaching to ~ 0.94 of appendix masculina.

###### Colouration.

Body colouration of large females whitish, frequently with broad bright red stripes and blotches, eggs green (Fig. 3A, B), males mostly transparent with some white blotches (Fig. 2E).

###### Reproductive biology and larval development.

Ovigerous females with few eggs (36, n = 1). Size of undeveloped eggs (early stage embryos without eyespot) 0.71–0.78 × 0.39–0.54 mm, size of developed eggs (late stage embryos with eyes) 0.78 × 0.47 mm (n = 9).

###### Etymology.

Named after the fourth author's, daughter for her strong interest in decapod crustaceans her father is working on.

###### Distribution.

*Caridina mayamareenae* sp. nov. is endemic to Lake Poso. Specimens were found at five localities within the lake, three in the northern part and two at the eastern and western shores in the southern part of the lake.

###### Ecology.

*Caridina mayamareenae* sp. nov. is hiding inside empty shells of the viviparid snail *Celetaia persculpta* (P. Sarasin and F. Sarasin, 1898) and *Tylomelania* spp. (Fig. 3D), and was not observed on any other substrate. On average, 1.4 shrimps were found per shell, but there is considerable variation (0.6–2.4 shrimps per shell) among the examined sites (Table 2). Up to four specimens were found in a single shell at the Dolidi Ndano locality. *Caridina mayamareenae* sp. nov. is also confined to deeper water; shells from depths of 7 m upwards did not contain any shrimps.

**Table 2.** Abundance of *Caridina mayamareenae* sp. nov. in shells of aquatic snails in Lake Poso. The numbers in brackets in the “shells” column refer to numbers of shells of *Celetaia persculpta* / shells of *Tylomelania* spp.; in the “other taxa” column, the numbers are juvenile gecarcinucid crabs / n *Cirolana* spp.

Locality and depth	shells (n)	shrimps (n)	Other taxa
E shore:			
Dolidi Ndano, 15m	28^*^	66	-^#^
W shore:			
Bay N of Cape Bancea, 15m	22 (13/9)	13	6/2
NW shore:			
Cape Wotu			
10m	25 (16/9)	30	-/1
15m	27 (27/0)	35	4/8
Siuri, 13–14m	19 (4/15)	23	4/-

Key: ^*^ total snail count, not differentiated between genera; ^#^ other taxa present, but not recorded.

###### Remarks.

Among all species of the genus *Caridina* known from Lake Poso, *C. mayamareenae* sp. nov. is unique by its short and conspicuous high rostrum, the less developed chelipeds with scarce setae at the tip of the fingers, and the strong third pair of pereiopods. A high and rather short rostrum is an infrequent character among lacustrine species of the genus *Caridina* from the Central Lakes of Sulawesi (compare revision in von Rintelen and Cai 2009). Many lacustrine species are showing slender, styliform rostrum shapes as seen in *C. ensifera* and *C. caerulea*, the most common species in Lake Poso. The conspicuous high rostrum and the strong third pair of pereiopods adapted for clinging on hard substrate are visible characters of a high grade of specialisation to the microhabitat of this species.

##### Caridina poso

Taxon classificationAnimaliaDecapodaAtyidae

Klotz, Wowor & K. von Rintelen sp. nov.

2EC26236-B29F-5BAC-B29D-B5CE168AF7B5

http://zoobank.org/83931AF8-E252-4068-94D9-69C1103D42D7

**Figure 13.**
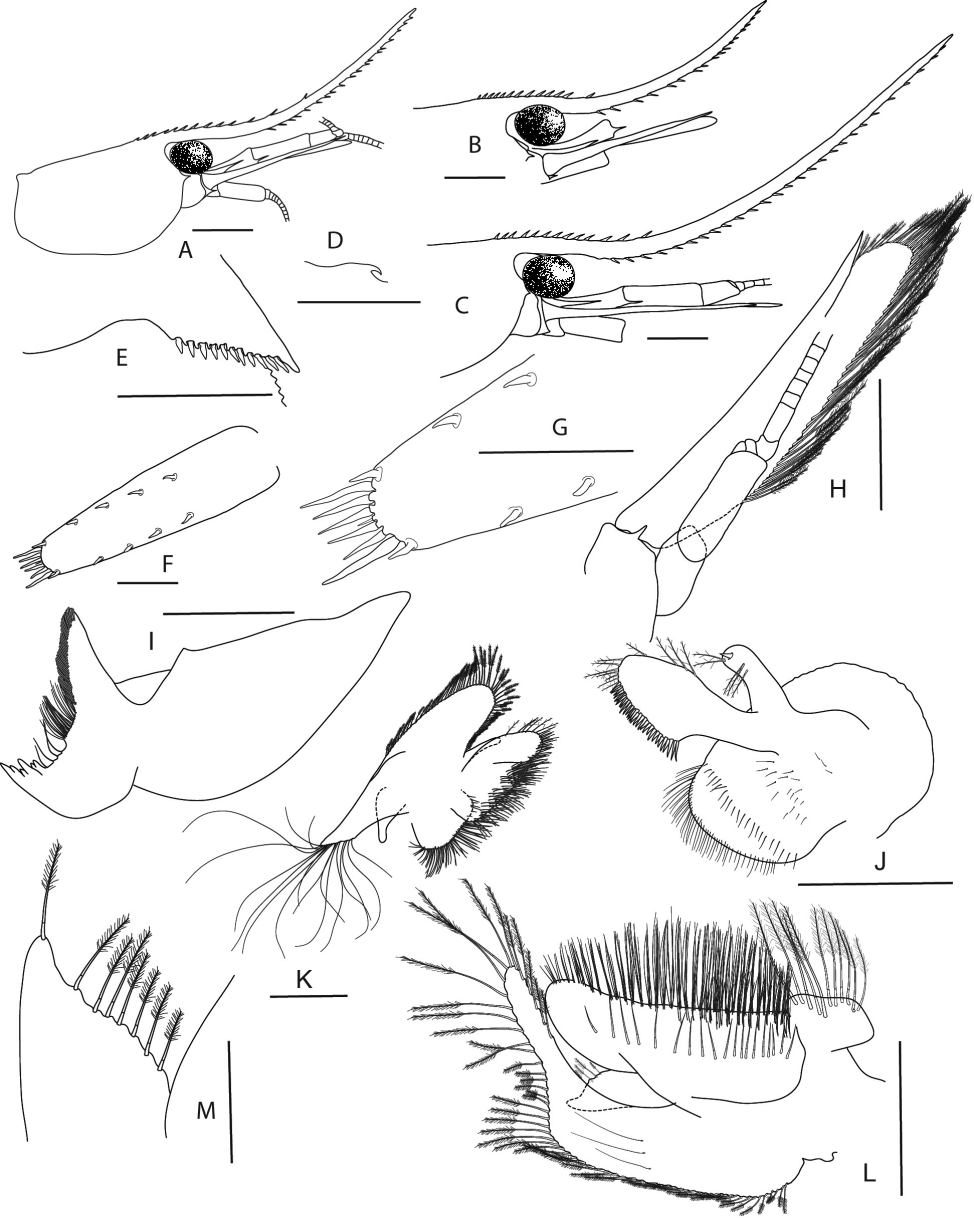
*Caridina poso* sp. nov. Morphology 1. ♂, cl. 2.7 mm, ZMB 29766 **A** cephalothorax and cephalic appendages **D** preanal carina **E** uropodal diaeresis **F, G** telson; paratype ♂, cl. 2.7 mm, ZMB 29624 **B** rostrum **H** scaphocerite **I** mandible **J** maxillula **K** maxilla **L** first maxilliped **M** palp of first maxilliped; paratype ov. ♀, cl. 3.4 mm, MZB Cru 5051 **C** Rostrum. Scale bars: 1 mm (**A–C, F, H, I**); 0.5 mm (**D, E, G, J–L**); 0.1 mm (**M**).

**Figure 14.**
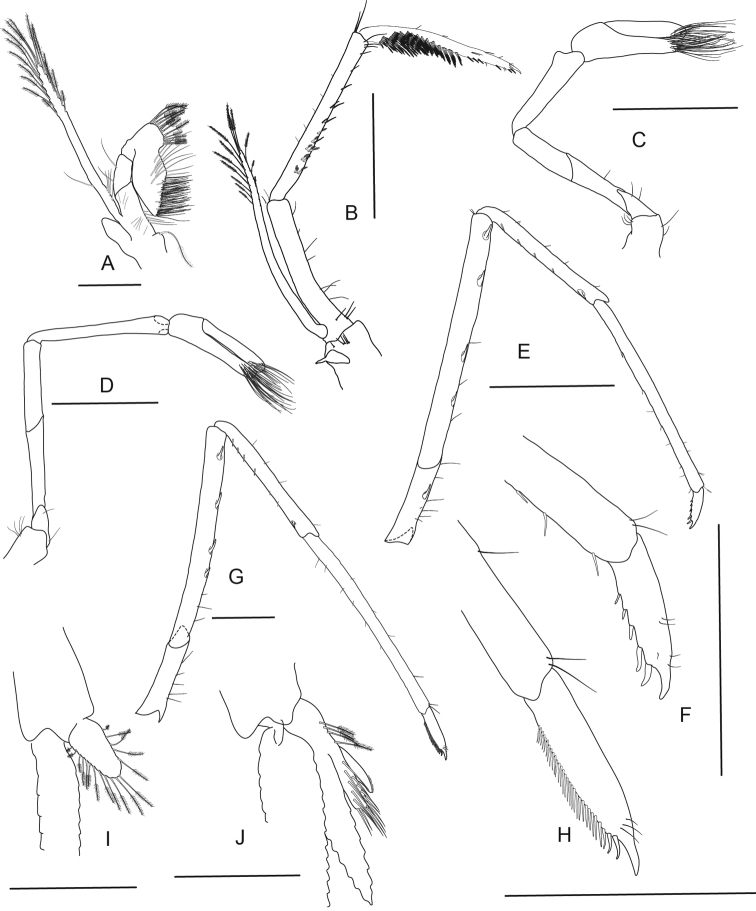
*Caridina poso* sp. nov. Morphology 2. Paratype ♂, cl. 2.7 mm, ZMB 29624 **A** second maxilliped **B** third maxilliped **C** first pereiopod **D** second pereiopod **E** third pereiopod **F** dactylus of third pereiopod **G** fifth pereiopod **H** dactylus of fifth pereiopod; ♂, cl. 2.7 mm, ZMB 29766 **I** endopod of male first pleopod **J** appendix masculina on male second pleopod. Scale bars: 0.5 mm (**A, F–J**); 1 mm (**B–E**).

Figures 3E–F , 13 , 14

###### Material examined.

***Holotype***: ov. ♂ cl. 2.7 mm (MZB Cru 5050), Indonesia, Central Sulawesi, Lake Poso, at Taipa, H-G. Evers leg., 26 Sep. 2010. **Paratypes**: 2 ov. ♀♀ cl. 2.9 and 3.1 mm, 3 ♀♀ cl. 3.0–3.1 mm, 2 ♂♂ cl. 2.6 and 2.7 mm (ZMB 29624), same data as holotype; 5 ov. ♀♀ cl. 3.2–3.4 mm, 5 ♂♂ cl. 2.7–3.2 mm (MZB Cru 5051), 5 ov. ♀♀ cl. 3.3–3.8 mm, 2 ♂♂ cl. 3.1 and 3.2 mm (ZMB 29621), Lake Poso, E shore, “Sulawesi Rock”, 1°56.102'S, 120°40.402'E, J. Pfaender and T. von Rintelen leg., 22 Sep. 2015; 5 ♂♂ cl. 2.4–2.7 mm (MZB Cru 5052), 1 ov. ♀ cl. 3.1 mm, 4 ♂♂ cl. 2.6–2.9 mm (ZMB 28063), Lake Poso, W shore, 1°56.67'S, 120°33.925'E, B. Stelbrink leg., 07 Sep. 2012.

***Other material***: 1 ♂ cl. 2.7 mm (ZMB 29766), aquarium reared specimen, preserved on 08 Oct. 2015 by W. Klotz.

###### Description.

***Cephalothorax and cephalic appendages.*** Postorbital carapace length 2.6–3.8 mm (n = 36). Rostrum (Fig. 13A-C) very long and slender, curved upwards, reaching far beyond end of scaphocerite, distal 0.5 to 0.8 unarmed, ventral margin armed throughout, most proximal tooth placed below third tooth of dorsal margin in most specimens, 1.35–2.75 (median 2.01, n = 23) times as long as carapace, rostral formula 3–5(4) + 8–14 / 19–37. Orbital margin fused with an antennal spine. Pterygostomial angle broadly rounded. Eyes well developed with globular cornea. Antennular peduncle (Fig. 13A, C), 0.97–1.03 (median 1.01, n = 4) times as long as carapace in females, 1.07–1.19 (median 1.16, n = 4) times as long as carapace in males, first segment 1.48–1.78 (median 1.55, n = 5) times as long as second segment, second segment 2.25–2.88 (median 2.44, n = 5) times longer than third segment. Stylocerite reaching to 0.78–0.88 (median 0.83, n = 4) of first segment of antennular peduncle. Scaphocerite (Fig. 13H) 4.30–5.33 (median 4.75, n = 6) times as long as wide.

***Abdominal somites, telson and uropods.*** Sixth abdominal somite 0.60–0.76 (median 0.67, n = 12) times carapace length, 1.70–2.18 (median 1.95, n = 11) times as long as fifth somite, 0.93–1.11 (median 1.08, n = 9) times as long as telson. Telson (Fig. 13F, G) 3.19–3.83 (median 3.50, n = 3) times as long as proximal wide, 5.73–6.42 (median 6.13, n = 3) times as long as distal wide, distal margin convex without a median projection, with three or four pairs of short spiniform setae dorsally and one pair of short spiniform setae dorsolaterally; distal end with 6–8 long spiniform setae, lateral pair slightly longer than others, innermost pair very tiny. Preanal carina (Fig. 13D) with a hook-like spine. Uropodal diaeresis (Fig. 13E) with 10–12 short movable spiniform setae, outermost ones shorter than lateral angle.

***Mouthparts and branchiae.*** Incisor process of mandible (Fig. 13I) ending in irregular teeth, molar process truncated. Lower lacinia of maxillula (Fig. 13J) broadly rounded, upper lacinia elongate, with numerous distinct teeth on inner margin, palp slender with few pappose setae and one conical spiniform seta near tip. Upper endites of maxilla (Fig. 13K) subdivided, palp slender, scaphognathite tapering posteriorly, fringed with long, curved setae at posterior margin. End of palp of first maxilliped triangular (Fig. 13L, M). Podobranch on second maxilliped (Fig. 14A) reduced to a lamina. Third maxilliped (Fig. 14B) with two arthrobranchs, ultimate segment of maxilliped shorter than penultimate segment. First pereiopod with an arthrobranch. Pleurobranchs present on all pereiopods. Epipod vestigial on third maxilliped, absent from all pereiopods.

***Pereiopods*.** Chelae of first and second pereiopods (Fig. 14C, D) well developed; chela of first pereiopod 3.11–3.36 (median 3.13, n = 5) times as long as wide, 0.95–1.21 (median 1.13, n = 5) times length of carpus; tips of fingers (Fig. 14C) rounded, without hooks, with tufts of hairs near tip; dactylus 2.00–2.50 (median 2.00, n = 5) times as long as palm; carpus slender, hardly excavated distally, 3.13–4.33 (median 3.76, n = 5) times as long as wide, 1.29–1.56 (median 1.45, n = 5) times length of merus. Merus 3.13–3.63 (median 3.38, n = 5) times as long as wide, 0.81–0.88 (median 0.85, n = 5) times as long as ischium. Chela of second pereiopod 3.23–3.91 (median 3.71, n = 6) times as long as wide, 0.67–0.84 (median 0.76, n = 6) times length of carpus; tips of fingers rounded, without hooks, with tufts of hairs near tip; dactylus 1.47–2.29 (median 2.00, n = 6) times as long as palm; carpus 6.50–8.38 (median 7.29, n = 6) times as long as wide, 1.38–1.63 (median 1.53, n = 6) times as long as merus; merus 5.08–6.43 (median 5.71, n = 6) times as long as wide, 0.86–0.96 (median 0.87, n = 6) times as long as ischium. Third pereiopod (Fig. 14E, F) not sexually dimorphic, dactylus 3.11–4.67 (median 3.86, n = 5) times as long as wide (terminal claw and spiniform setae on flexor margin included), terminating in one large claw with 5–7 short spiniform setae on flexor margin; propodus very slender, 16.50–19.11 (median 18.71, n = 6) times as long as wide, 4.78–6.00 (median 5.12, n = 6) times as long as dactylus; carpus 8.73–10.40 (median 9.50, n = 6) times as long as wide, 0.64–0.72 (median 0.70, n = 6) times as long as propodus; merus slender, 11.25–13.93 (median 12.31, n = 6) times as long as wide, 1.54–1.71 (median 1.66, n = 6) times as long as carpus, bearing 4 strong spiniform setae on posterior margin of outer surface. Ischium with one spiniform seta. Fifth pereiopod (Fig. 14G, H) slender, dactylus 2.43–4.67 (median 3.69, n = 6) times as long as wide (terminal claw and serrate setae on flexor margin included), terminating in one large claw, with 30–34 serrate setae on flexor margin; propodus slender, 15.80–21.50 (median 16.97, n = 6) times as long as wide, 4.41–5.28 (median 4.73, n = 6) times length of dactylus, carpus 7.27–10.40 (median 8.30, n = 6) times as long as wide, 0.51–0.61 (median 0.59, n = 6) times as long as propodus; merus slender, 11.50–12.92 (median 11.74, n = 6) times as long as wide, 1.35–1.63 (median 1.50, n = 6) times length of carpus, bearing four or five strong spiniform setae on posterior margin of outer surface. Ischium without a strong spiniform seta.

***Pleopods*.** Endopod of male first pleopod (Fig. 14I) subtriangular, without an appendix interna, 1.77–2.27 (median 1.83, n = 3) times as long as proximal width, 0.21–0.27 (median 0.23, n = 3) times as long as exopod. Appendix masculina on male second pleopod (Fig. 14J) slender, rod-shaped, 7.25–10.50 (median 7.67, n = 3) times as long as wide, with long spiniform setae on inner and distal margin, a few pappose setae on basal part, appendix interna reaching to ~ 0.65–0.90 (median 0.85, n = 3) of appendix masculina.

###### Colouration.

Body and legs mottled with reddish and white dots arranged in rows, exopod of uropods with a black and white blotch, antennae dark red, chelae white with red fingers (Fig. 3E, F).

###### Reproductive biology and larval development.

Ovigerous females with few, large eggs (5 and 9, n = 2). Size of eggs 0.96–1.11 × 0.56–0.66 mm (n = 9).

###### Etymology.

The specific name is a noun in apposition after the type locality, Lake Poso.

###### Distribution.

*Caridina poso* sp. nov. is endemic to Lake Poso. Specimens were found at three localities within the lake, one at the east shore and two at the west shore.

###### Ecology.

*Caridina poso* sp. nov. lives in packs of debris (small to medium-sized stones) close to the shore of the Lake Poso and thus could be considered a hard substrate dweller as defined in von Rintelen and Cai (2009). The species was never found on soft substrates such as dead leaves, wood or water plants.

###### Remarks.

With its long and upturned rostrum, *C. poso* sp. nov. is similar to *C. ensifera* and *C. caerulea*, two endemic species to Lake Poso. In the field, colouration alone is sufficient to differenciate *C. poso* sp. nov. from these species. The much smaller species *C. poso* sp. nov. (carapace length 2.6–3.8 mm) is showing black and white blotches on the exopod of the uropods. In the larger species *C. ensifera* (cl. 3.5–5.3 mm), a dark red spot is seen on the exopod of the uropods. In *C. caerulea* (cl. 3.0–4.5 mm), the exopod of the uropods shows an elongate blue blotch (von Rintelen and Cai 2009).

In preserved condition, *C. poso* sp. nov. can be distinguished from *C. ensifera* by the absence of epipods on all pereiopods (a vestigial epipod is present on third maxilliped vs. epipods well developed, with distal hooks on the third maxilliped and first and second pereiopods in *C. ensifera*) and by the higher number of postorbital teeth on the rostrum (3–5 (mode 4) vs. 1–3 (mode 2) in *C. ensifera*). Further, *C. poso* sp. nov. differs by its slender chelipeds (chela of first pereiopod 3.1–3.4 times as long as high vs. 2.0–2.8 times in *C. ensifera*, dactylus of first cheliped 2.0–2.5 times as long as palm vs. 1.0–1.3 times in *C. ensifera*, dactylus of second cheliped 1.5–2.3 times as long as palm vs. 1.2–1.4 times in *C. ensifera*) and slender third pair of pereiopods (propodus 16.5–19.1 times as long as wide vs. 10–13 times in *C. ensifera*, carpus 8.7–10.4 times as long as wide vs. 4.7–6.1 times in *C. ensifera*, merus 11.3–13.9 times as long as wide vs. 9.2–11.4 times in *C. ensifera*). In contrast, the dactyli of the fifth pereiopods are shorter (dactylus 2.4–4.7 times as long as wide vs. 5.4–7.0 times in *C. ensifera*, propodus 4.4–5.3 times as long as dactylus vs. 2.5–3.0 times in *C. ensifera*). The dactyli of fifth pereiopods are armed with a smaller number of serrate setae on the flexor margin (30–34 vs. 51–57 in *C. ensifera*). *Caridina poso* sp. nov. differs from *C. caerulea* by the absence of epipods on all pereiopods (a vestigial epipod is present on the third maxilliped) vs. epipods well developed (with distal hooks) on the third maxilliped and first and second pereiopods and the higher number of postorbital teeth on the rostrum (3–5 (mode 4) vs. 2–4 (mode 2)) in *C. caerulea*; further by its shorter telson (telson 0.6–0.7 times as long as carapace vs. 0.8 times in *C. caerulea*), by the slender chelipeds (chela of first pereiopod 3.1–3.4 times as long as high vs. 1.9–2.7 times in *C. caerulea*, dactylus of first cheliped 2.0–2.5 times as long as palm vs. 1.1–1.4 times in *C. caerulea*, carpus of first cheliped 3.1–4.3 times as long as wide vs. 2.1–2.5 times in *C. caerulea*; chela of second cheliped 3.2–3.9 times as long as wide vs. 2.1–3.2 times in *C. caerulea*, dactylus of second cheliped 1.5–2.3 times as long as palm vs. 1.3–1.6 times in *C. caerulea*, carpus of second cheliped 6.5–8.4 times as long as wide vs. 4.1–5.4 times in *C. caerulea*). The third pair of pereiopods is more slender (propodus 16.5–19.1 times as long as wide vs. 12.9–16.3 times in *C. caerulea*, carpus 8.7–10.4 times as long as wide vs. 5.9–8.0 times in *C. caerulea*, merus 11.3–13.9 times as long as wide vs. 9.4–11.8 times in *C. caerulea*). Merus of fifth pereiopod slender, 11.5–12.9 times as long as wide vs. 8.5–11.3 times in *C. caerulea*.

### Molecular phylogenetics

We used sequences of mitochondrial DNA to investigate the phylogenetic relationship among the species of *Caridina* from Lake Poso as described above. The resulting sequence alignments have a length of 781 bp (COI) and 540 bp (16S), respectively. In 16S, only two short and largely unambiguous indels (1–2 bp) were required to homologise positions in the alignment.

If support values are considered, the tree topologies reconstructed from 16S and COI are largely congruent (Suppl. material 2, Figs S4, S5). All well supported clades (BPP > 0.9) are found in both trees, while basal splits of both trees, particularly for 16S, are poorly supported.

**Figure 15.**
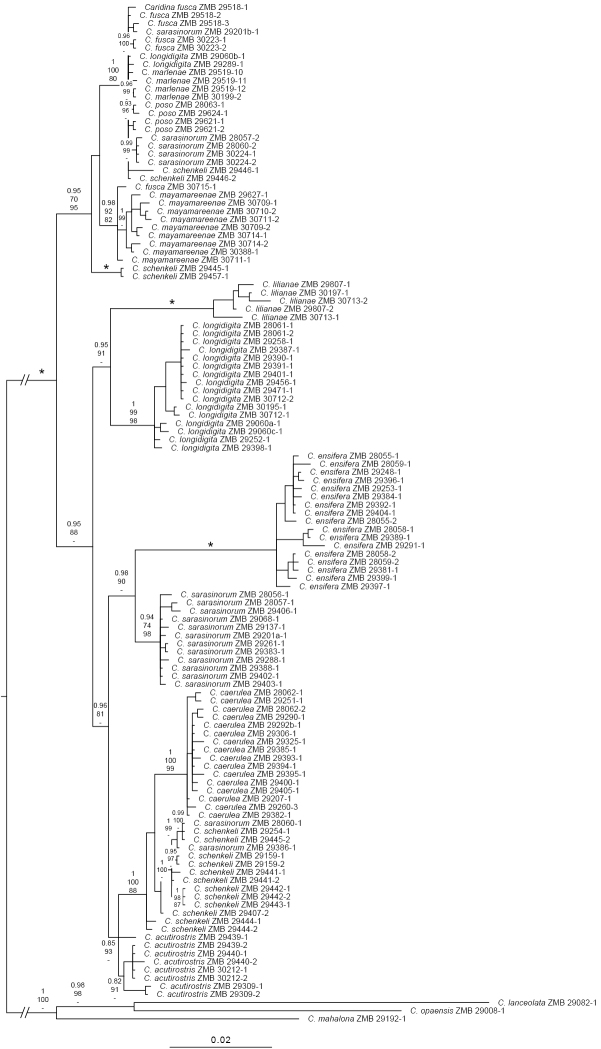
Molecular phylogeny of the 11 species of *Caridina* in Lake Poso. Phylogenetic relationships reconstructed by BI analyses of two mitochondrial gene fragments (topology based on concatenated 16S and COI datasets). Number of branches show, from top, Bayesian posterior probabilities (> 0.85) and ML/MP bootstrap values (> 70). An asterisk indicates nodes with full support (1/100/100). The scale bar indicates the substitution rate. See Suppl. material 1 for information on the sequenced specimens.

The molecular phylogeny of Lake Poso species for this study and from the previous study with fewer species (von Rintelen et al. 2007a) revealed similar results: a) All 11 species from the lake as well as its catchment area (Table 1) form a well-supported monophyletic group (Fig. 15; Suppl. material 2: Figs S1–S3); b) the nine species from the lake proper do not form a monophylum but cluster in separate groups as highlighted in Figure 15, partly clustering with the riverine species *Caridina acutirostris* and *C. schenkeli*; c) the match of morphospecies and genetic clades remains as heterogeneous as in von Rintelen et al. (2007a). Only four of the eleven morphospecies consistently correspond to mtDNA clades (*C. acutirostris*, *C. caerulea*, *C. ensifera*, and *C. lilianae* sp. nov.). *Caridina mayamareenae* sp. nov. also forms a single clade but contains one specimen of *C. fusca* sp. nov. Two more species (*Caridina longidigita* and *C. sarasinorum*) form distinct clades comprising the majority of sequenced specimens, but not all. A few specimens of these two species are also found within a clade comprising all sequences of *C. marlenae* sp. nov. and *C. poso* sp. nov. as well as several specimens of *C. sarasinorum, C. schenkeli* and the second known population of *C. fusca* sp. nov. The majority of *C. schenkeli* specimens form a paraphyletic group with respect to *C. caerulea*, and this group also includes one sequence of *C. sarasinorum*.

## Discussion

### Lake Poso revisited – new insights from new material?

The molecular phylogeny and field observations (colour pattern, habitat, distribution, behaviour if applicable) were used to test and support the morphological studies of alcohol preserved specimens. The integrative taxonomic approach taken by von Rintelen et al. (2007a) and von Rintelen and Cai (2009) was again successfully applied in this study.

The match of morphospecies and genetic clades remains as heterogeneous as in von Rintelen et al. (2007a). Although all new species are morphologically distinguishable based on the characters described in this study, only four species form exclusively monophyletic groups (Fig. 15). The non-monophyly of the remaining species including three of the new species described here might be explained by introgressive hybridisation or incomplete lineage sorting as discussed for the previously described species. It remains to be seen whether the hypothesis forwarded by von Rintelen et al. (2007a) and von Rintelen and Cai (2009) that the colour patterns of the hybridising species seem to be less obvious and stable than those of the monophyletic taxa, occasionally resulting in mating errors between lake species and between riverine and lake species, holds true. Among the newly described species, *Caridina poso* sp. nov. and *C. marlenae* sp. nov. show characteristic, stable, and in the latter also rather conspicuous colour patterns. This hypothesis, however, needs further testing. Another assumption explaining the insufficient resolution by molecular data in most of the new species, which possibly also led to their late discovery, is that their species boundaries are not as fixed yet and they are still in the process of becoming proper biological species. A similar case is known from four closely related *Caridina* species from Lake Towuti, Sulawesi (von Rintelen and Cai 2009). In a future study, this assumption could be tested and distinguished from introgressive hybridisation by applying a population genomics approach such as, e.g., RAD seq, which has been applied to much the same purpose in Lake Poso ricefishes (Sutra et al. 2019).

### Adaptive radiation in Lake Poso

Von Rintelen et al. (2007a) positively tested the hypothesis of an adaptive radiation in the atyid shrimp species flock in Lake Poso, which met at least three of the four criteria defined by Schluter (2000): All eleven species, including those from the catchment area, showed common ancestry, indicative of one colonisation of the entire Poso lake system, and rapid radiation. Apart from the well supported monophyly of the entire species flock, the non-monophyly of the nine lake species are congruent with the results found by von Rintelen et al. (2007a). This time again, the data failed to provide conclusive evidence for an *in situ* radiation within the lake itself. The earlier study revealed a correlation of phenotype and environment (habitat preferences and divergence in trophic morphology) in Lake Poso species. By exhibiting species-specific and unusual colour patterns, the species seemed to have reached the third stage of adaptive radiation according to Streelman and Danley (2003), i.e., sexual selection suggested as a driving force of diversification (see review in von Rintelen et al. 2020).

The new species cluster within the Poso clade (Fig. 15) are thus part of the monophyletic species flock (common ancestry) that radiated into several specialised ancestors. All new lake species also show pronounced microhabitat preferences – the most extreme example is *Caridina mayamareenae* sp. nov. (Fig. 3A, B) – along with interspecific differences in cheliped morphology (Figs 6, 8, 10, 12, 14) and species-specific colour patterns (Figs 2E, 3A, B), although not always as clear as described in von Rintelen and Cai (2009). The adaptive radiation of shrimps in Lake Poso is thus more diverse than previously assumed, not just in terms of species number, but also with respect to habitat and trophic specialisation. A parallel case of adaptive and largely microhabitat-driven radiation in *Caridina* is know from the Malili lake system of Sulawesi (von Rintelen et al. 2010; Martin and Richards 2019).

### Atyid shrimps in association with other organisms

The occurrence of a Lake Poso species in empty snail shells (i.e., *Caridina mayamareenae* sp. nov.; Fig. 3A, B, D) is rather unusual, as empty shells of aquatic snails were not reported as microhabitat of atyid shrimp up to now.

In Lake Tanganyika, East Africa, Roth-Woltereck (1958) described the small atyid species *Limnocaridina iridinae* based on two ovigerous females found in the gill chamber of the bivalve *Iridina spekei*. Later, no further specimen of this shrimp species was found. Only in November 2010, the first author received a single ovigerous specimen of *L. iridinae* (now deposited in the crustacean collection of the Oxford University Museum of Natural History, collection number OUMNH.ZC.2012-05-0012) for determination. This time, it was found in an empty shell of the viviparid snail *Neothauma tanganyicense*. According to the collector, approximately 100 specimens of *Iridina spekei* were checked for specimens of shrimp without any success (Heinz Büscher, pers. comm.). This might indicate that *L. iridinae* is not only associated with bivalves but likewise seeks shelter in empty snail shells.

So far, *Limnocaridina iridinae* and the parallel case of *C. mayamareenae* sp. nov. from Lake Poso are the only cases of freshwater shrimps in general, and particularly in ancient lake species flocks, associated with molluscs. As a possibly morphological adaptation to their habitat, both species share the less developed type of chelae bearing just some scarce setae at the tips of fingers in contrast to the brush-like dense tufts of setae found in other atyids. A similar association was only described for *C. spongicola* from the Malili lake system, Sulawesi. This species is associated with an endemic freshwater sponge from Lake Towuti and one of the most extreme specialisations found in the adaptive radiation of *Caridina* in the ancient lakes of Sulawesi (Zitzler and Cai 2006; von Rintelen et al. 2007b). All three cases might be seen as an example of ecological convergence between species of all three ancient lake systems.

### Conservation status and sustainability

Following the IUCN categories, all previously described species from Lake Poso and catchment (Table 1) were assessed as Vulnerable under the D2 criterion (De Grave et al. 2013a–e, Wowor et al. 2013). This was justified based on the limited occurrence of endemic populations and the presence of an introduced fish species as a plausible threat. This originally African cichlid species was again observed in Lake Poso in 2019 (KvR, TvR pers. obs.). The five new *Caridina* species are thus subjected to similar threats and have a similarly limited occurrence in the lake system as the previously described species. We therefore suggest to include the new species under the same IUCN category and criterion. Currently, the third author and Indonesian colleagues are preparing measures to protect the habitats and fauna of the ancient lakes of Sulawesi, including the atyid species flocks from Lake Poso and the Malili lakes summarised in this study and in von Rintelen and Cai (2009).

The key to pre-sorting living specimens in the field can be used easily without having to use microscopic equipment. Shrimps can, for example, be observed while swimming or snorkelling or by putting them in small fish tanks, and releasing back into the water afterwards. The key can be used for pre-sorting in the field for scientific purpose but also for sustainable capacity building or citizen science projects without having to reduce the populations. This key, however, has not fully been tested in the field and would certainly be an ideal test case for a local citizen science or student project.

## Conclusions

Even in relatively well studied areas like the ancient lakes of Sulawesi, the biodiversity of freshwater shrimps has largely been underestimated. An integrative taxonomic approach is the key to the discovery of new species and to a better understanding of the evolution of Lake Poso's fauna. This new knowledge can contribute to the prevention of biodiversity and habitat loss.

## Supplementary Material

XML Treatment for Caridina fusca

XML Treatment for Caridina lilianae

XML Treatment for Caridina marlenae

XML Treatment for Caridina mayamareenae

XML Treatment for Caridina poso

## Footnotes

This key should not be used to identify specimens bred in captivity. Based on occasional colour hybrids not occurring in the natural environment, it could produce misleading results.

## Supplementary materials

Supplementary material 1 Table S1. Sample provenience and accession numbers Data type molecular data https://binary.pensoft.net/file/493873This dataset is made available under the Open Database License (http://opendatacommons.org/licenses/odbl/1.0/). The Open Database License (ODbL) is a license agreement intended to allow users to freely share, modify, and use this Dataset while maintaining this same freedom for others, provided that the original source and author(s) are credited.Werner Klotz, Thomas von Rintelen, Daisy Wowor, Chris Lukhaup, Kristina von Rintelen

Supplementary material 2 Supplementary figures Data type multimedia Explanation note **Figure S1.** Phylogenetic relationships reconstructed by BI analyses of two mitochondrial gene fragments (topology based on concatenated 16S and COI datasets). The scale bar indicates the substitution rate. Original Bayesian posterior probabilities of Fig. 13. **Figure S2.** Phylogenetic relationships reconstructed by ML analyses of two mitochondrial gene fragments (topology based on concatenated 16S and COI datasets). The scale bar indicates the substitution rate. **Figure S3.** Phylogenetic relationships reconstructed by MP analyses of two mitochondrial gene fragments (topology based on concatenated 16S and COI datasets). **Figure S4.** Phylogenetic relationships reconstructed by ML analyses of one mitochondrial gene fragments (16S). The scale bar indicates the substitution rate. **Figure S5.** Phylogenetic relationships reconstructed by ML analyses of one mitochondrial gene fragments (COI). The scale bar indicates the substitution rate. https://binary.pensoft.net/file/493874This dataset is made available under the Open Database License (http://opendatacommons.org/licenses/odbl/1.0/). The Open Database License (ODbL) is a license agreement intended to allow users to freely share, modify, and use this Dataset while maintaining this same freedom for others, provided that the original source and author(s) are credited.Werner Klotz, Thomas von Rintelen, Daisy Wowor, Chris Lukhaup, Kristina von Rintelen

## References

[B1] Cai Y Wowor D (2007) Atyid shrimps from Lake Poso, Central Sulawesi, Indonesia with description of a new species (Crustacea: Decapoda: Caridea). The Raffles Bulletin of Zoology 55: 311–320.

[B2] Chernomor O von Haeseler A Minh BQ (2016) Terrace aware data structure for phylogenomic inference from supermatrices. Systematic Biology 65(6): 997–1008. 10.1093/sysbio/syw037 27121966 PMC5066062

[B3] Coleman CO (2003) “Digital inking”: How to make perfect line drawings on computers. Organisms Diversity & Evolution 3(4): 1–14. 10.1078/1439-6092-00081

[B4] Coleman CO (2006) Substituting time-consuming pencil drawings in arthropod taxonomy using stacks of digital photographs. Zootaxa 1360: 61–68. 10.11646/zootaxa.1360.1.4

[B5] De Grave S Wowor D Klotz W (2013a) *Caridina acutirostris*. The IUCN Red List of Threatened Species 2013. e.T197725A2497568.

[B6] De Grave S Wowor D Klotz W (2013b) *Caridina caerulea*. The IUCN Red List of Threatened Species 2013. e.T197973A2507226.

[B7] De Grave S Wowor D Klotz W (2013c) *Caridina ensifera*. The IUCN Red List of Threatened Species 2013: e.T197967A2506841.

[B8] De Grave S Wowor D Klotz W (2013d) *Caridina sarasinorum*. The IUCN Red List of Threatened Species 2013: e.T198277A2518617.

[B9] De Grave S Wowor D Klotz W (2013e) *Caridina schenkeli*. The IUCN Red List of Threatened Species 2013: e.T197587A2491852.

[B10] Hoang DT Chernomor O von Haeseler A Minh BQ Vinh LS (2018) UFBoot2: Improving the ultrafast bootstrap approximation. Molecular Biology and Evolution 35: 518–522. 10.1093/molbev/msx281 29077904 PMC5850222

[B11] Huelsenbeck JP Ronquist F Nielsen R Bollback JP (2001) Bayesian inference of phylogeny and its impact on evolutionary biology. Science, 294: 2310–2314. 10.1126/science.1065889 11743192

[B12] Katoh K Standley DM (2013) MAFFT multiple sequence alignment software version 7: improvements in performance and usability. Molecular Biology and Evolution 30: 772–780. 10.1093/molbev/mst010 23329690 PMC3603318

[B13] Kumar S Stecher G Li M Knyaz C Tamura K (2018) MEGA X: Molecular evolutionary genetics analysis across computing platforms. Molecular Biology and Evolution 35: 1547–1549. 10.1093/molbev/msy096 29722887 PMC5967553

[B14] Lai H-T Shy J-Y (2009) The larval development of *Caridina pseudodenticulata* (Crustacea: Decapoda: Atyidae) reared in the laboratory, with a discussion of larval metamorphosis types. The Raffles Bulletin of Zoology, Supplement 20: 97–107.

[B15] Martin CH Richards EJ (2019) The paradox behind the pattern of rapid adaptive radiation: how can the speciation process sustain itself through early burst? Annual Review of Ecology, Evolution, and Systematics 50: 569–593. 10.1146/annurev-ecolsys-110617-062443 PMC9555815 36237480

[B16] Nguyen LT Schmidt HA von Haeseler A Minh BQ (2015) IQ-TREE: A fast and effective stochastic algorithm for estimating maximum likelihood phylogenies. Molecular Biology and Evolution 32: 268–274. 10.1093/molbev/msu300 25371430 PMC4271533

[B17] Posada D (2008) jModelTest: phylogenetic model averaging. Molecular Biology and Evolution 25(7): 1253–1256. 10.1093/molbev/msn083 18397919

[B18] Ronquist F Teslenko M van der Mark P Ayres DL Darling A Höhna S Larget B Liu L Suchard MA Huelsenbeck JP (2012) MrBayes 3.2: efficient Bayesian phylogenetic inference and model choice across a large model space. Systematic Biology 61: 539–542. 10.1093/sysbio/sys029 22357727 PMC3329765

[B19] Roth-Woltereck E (1958) *Limnocaridina iridinae*, n. sp. Eine interessante Garnele aus dem Tanganijka-See (DecapodaAtyidae). Zoologischer Anzeiger 161: 188–192.

[B20] Schenkel E (1902) Beitrag zur Kenntnis der Dekapodenfauna von Celebes. Verhandlungen der Naturforschenden Gesellschaft in Basel 13: 485–585.

[B21] Schluter D (2000) The Ecology of Adaptive Radiation. Oxford University Press, Oxford, 296 pp.

[B22] Short JW (2004) A revision of Australian river prawns, *Macrobrachium* (Crustacea: Decapoda: Palaemonidae. Hydrobiologia 525: 1–100. 10.1023/B:HYDR.0000038871.50730.95

[B23] Streelman JT Danley PD (2003) The stages of vertebrate evolutionary radiation. Trends in Ecology & Evolution 18: 126–131. 10.1016/S0169-5347(02)00036-8

[B24] Sutra N Kusumi J Montenegro J Kobayashi H Fujimoto S Masengi KWA Nagano AJ Toyoda A Matsunami M Kimura R Yamahira K (2019) Evidence for sympatric speciation in a Wallacean ancient lake. Evolution 73: 1898–1915. 10.1111/evo.13821 31407798

[B25] Swofford DL (2002) PAUP* (version 4.0). Phylogenetic analysis using parsimony (*and other methods). Sinauer Associates, Sunderland, Massachusetts, USA .

[B26] Vaillant JJ Haffner GD Cristecu ME (2011) The ancient lakes of Indonesia: towards integrated research on speciation. Integrative and Comparative Biology 51(4): 634–643. 10.1093/icb/icr101 21856735

[B27] von Rintelen K Cai Y (2009) Radiation of endemic species flocks in ancient lakes: Systematic revision of the freshwater shrimp *Caridina* H. Milne Edwards, 1837 (Crustacea: Decapoda: Atyidae) from the ancient lakes of Sulawesi, Indonesia, with the description of eight new species. The Raffles Bulletin of Zoology 57: 343–452.

[B28] von Rintelen K von Rintelen T Glaubrecht M (2007a) Molecular phylogeny and diversification of freshwater shrimps (Decapoda, Atyidae, *Caridina*) from ancient Lake Poso (Sulawesi, Indonesia) – the importance of being colourful. Molecular Phylogenetics and Evolution 45: 1033–1041. 10.1016/j.ympev.2007.07.002 17702608

[B29] von Rintelen K von Rintelen T Meixner M Lüter C Cai Y Glaubrecht M (2007b) Freshwater shrimp-sponge association from an ancient lake. Biology Letters 3: 262–264. 10.1098/rsbl.2006.0613 17347103 PMC2464681

[B30] von Rintelen K Glaubrecht M Schubart CD Wessel A von Rintelen T (2010) Adaptive radiation and ecological diversification of Sulawesi's, ancient lake shrimps. Evolution 64: 3287–99. 10.1111/j.1558-5646.2010.01043.x 20500216

[B31] von Rintelen T von Rintelen K Glaubrecht M Schubart CD Herder F (2012) Aquatic biodiversity hotspots in Wallacea: the species flocks in the ancient lakes of Sulawesi Indonesia. In: Gower DJ Johnson KG Richardson JE Rosen BR Rüber L Williams ST (Eds) Biotic Evolution and Environmental Change in Southeast Asia.Cambridge University Press, Cambridge, 290–315. 10.1017/CBO9780511735882.014

[B32] von Rintelen K De los Ríos P von Rintelen T (2020) Standing waters, especially ancient lakes. In: Poore GCB Thiel M (Eds) The Natural History of the Crustacea: Evolution and Biogeography, Volume 8.Oxford University Press, New York, 296–318.

[B33] Wowor D De Grave S Klotz W (2013) *Caridina longidigita*. The IUCN Red List of Threatened Species 2013: e.T197820A2501422.

[B34] Zitzler K Cai Y (2006) *Caridina spongicola*, new species, a freshwater shrimp (Crustacea: Decapoda: Atyidae) from the ancient Malili lake system of Sulawesi, Indonesia. The Raffles Bulletin of Zoology 54: 271–276.

