# Supplementary material for: Lake Poso's shrimp fauna revisited: the description of five new species of the genus Caridina (Crustacea, Decapoda, Atyidae) more than doubles the number of endemic lacustrine species
Source: Zookeys. 2021 Jan 4;1009:81–122. doi: 10.3897/zookeys.1009.54303 (PMC7801368; doi:10.3897/zookeys.1009.54303)
Supplement: Supplementary material 1 — Table S1. Sample provenience and accession numbers [file zookeys-1009-081-s001.docx]

**Supplementary material**

**Supplementary Table 1.** Sample provenience and accession numbers.

| **Species (ingroup)** | **Voucher No.** | **GenBank No.** | | **Source^*^** |
| --- | --- | --- | --- | --- |
|  |  | **COI** | **16S** |  |
| *Caridina acutirostris* | ZMB 29309-1 | AM747726 | AM747635 | von Rintelen et al. 2007 |
|  | ZMB 29309-2 | AM747727 | AM747636 | von Rintelen et al. 2007 |
|  | ZMB 29439-1 | AM747728 | AM747637 | von Rintelen et al. 2007 |
|  | ZMB 29439-2 | AM747729 | AM747638 | von Rintelen et al. 2007 |
|  | ZMB 29440-1 | AM747730 | AM747639 | von Rintelen et al. 2007 |
|  | ZMB 29440-2 | AM747731 | AM747640 | von Rintelen et al. 2007 |
|  | ZMB 30212-1 | MT769192 | MT769137 | This study |
|  | ZMB 30212-2 | MT769193 | MT769138 | This study |
| *Caridina caerulea* | ZMB 28062-1 | MT769194 | MT769139 | This study |
|  | ZMB 28062-2 | MT769195 | MT769140 | This study |
|  | ZMB 29207-1 | AM747732 | AM747650 | von Rintelen et al. 2007 |
|  | ZMB 29251-1 | AM747733 | AM747713 | von Rintelen et al. 2007 |
|  | ZMB 29260-3 | AM747735 | AM747651 | von Rintelen et al. 2007 |
|  | ZMB 29290-1 | AM747736 | AM747641 | von Rintelen et al. 2007 |
|  | ZMB 29292b-1 | AM747739 | AM747649 | von Rintelen et al. 2007 |
|  | ZMB 29306-1 | AM747740 | AM747652 | von Rintelen et al. 2007 |
|  | ZMB 29325-1 | AM747741 | AM747653 | von Rintelen et al. 2007 |
|  | ZMB 29382-1 | AM747743 | AM747642 | von Rintelen et al. 2007 |
|  | ZMB 29385-1 | AM747745 | AM747643 | von Rintelen et al. 2007 |
|  | ZMB 29393-1 | AM747748 | AM747644 | von Rintelen et al. 2007 |
|  | ZMB 29394-1 | AM747749 | AM747645 | von Rintelen et al. 2007 |
|  | ZMB 29395-1 | AM747750 | AM747646 | von Rintelen et al. 2007 |
|  | ZMB 29400-1 | AM747754 | AM747647 | von Rintelen et al. 2007 |
|  | ZMB 29405-1 | AM747756 | AM747648 | von Rintelen et al. 2007 |
| *Caridina ensifera* | ZMB 28055-1 | MT769196 | MT769141 | This study |
|  | ZMB 28055-2 | MT769197 | MT769142 | This study |
|  | ZMB 28058-1 | MT769198 | MT769143 | This study |
|  | ZMB 28058-2 | MT769199 | MT769144 | This study |
|  | ZMB 28059-1 | MT769200 | MT769145 | This study |
|  | ZMB 28059-2 | MT769201 | MT769146 | This study |
|  | ZMB 29248-1 | AM747737 | AM747655 | von Rintelen et al. 2007 |
|  | ZMB 29253-1 | AM747734 | AM747654 | von Rintelen et al. 2007 |
|  | ZMB 29291-1 | AM747738 | AM747664 | von Rintelen et al. 2007 |
|  | ZMB 29381-1 | AM747742 | AM747656 | von Rintelen et al. 2007 |
|  | ZMB 29384-1 | AM747744 | AM747657 | von Rintelen et al. 2007 |
|  | ZMB 29389-1 | AM747746 | AM747658 | von Rintelen et al. 2007 |
|  | ZMB 29392-1 | AM747747 | AM747659 | von Rintelen et al. 2007 |
|  | ZMB 29396-1 | AM747751 | AM747660 | von Rintelen et al. 2007 |
|  | ZMB 29397-1 | AM747752 | AM747661 | von Rintelen et al. 2007 |
|  | ZMB 29399-1 | AM747753 | AM747662 | von Rintelen et al. 2007 |
|  | ZMB 29404-1 | AM747755 | AM747663 | von Rintelen et al. 2007 |
| *Caridina fusca* | ZMB 29518-1 | MT769202 | MT769148 | This study |
|  | ZMB 29518-2 | MT769203 | MT769149 | This study |
|  | ZMB 29518-3 | MT769204 | MT769150 | This study |
|  | ZMB 30223-1 | MT769205 | MT769151 | This study |
|  | ZMB 30223-2 | MT769206 | MT769152 | This study |
|  | ZMB 30715-1 | MT769207 | MT769153 | This study |
| *Caridina lilianae* | ZMB 29807-1 | MT769208 | MT769154 | This study |
|  | ZMB 29807-2 | MT769209 | MT769155 | This study |
|  | ZMB 30197-1 | MT769210 | MT769156 | This study |
|  | ZMB 30713-1 | MT769211 | MT769157 | This study |
|  | ZMB 30713-2 | MT769212 | MT769158 | This study |
| *Caridina longidigita* | ZMB 28061-1 | MT769213 | MT769159 | This study |
|  | ZMB 28061-2 | MT769214 | MT769160 | This study |
|  | ZMB 29060a-1 | AM747771 | AM747674 | von Rintelen et al. 2007 |
|  | ZMB 29060b-1 | AM747772 | AM747675 | von Rintelen et al. 2007 |
|  | ZMB 29060c-1 | AM747773 | AM747667 | von Rintelen et al. 2007 |
|  | ZMB 29252-1 | AM747777 | AM747666 | von Rintelen et al. 2007 |
|  | ZMB 29258-1 | AM747779 | AM747677 | von Rintelen et al. 2007 |
|  | ZMB 29289-1 | AM747780 | AM747673 | von Rintelen et al. 2007 |
|  | ZMB 29387-1 | AM747784 | AM747668 | von Rintelen et al. 2007 |
|  | ZMB 29390-1 | AM747785 | AM747669 | von Rintelen et al. 2007 |
|  | ZMB 29391-1 | AM747786 | AM747670 | von Rintelen et al. 2007 |
|  | ZMB 29398-1 | AM747787 | AM747671 | von Rintelen et al. 2007 |
|  | ZMB 29401-1 | AM747788 | AM747672 | von Rintelen et al. 2007 |
|  | ZMB 29456-1 | AM747803 | AM747676 | von Rintelen et al. 2007 |
|  | ZMB 29471-1 | MT769215 | MT769161 | This study |
|  | ZMB 30195-1 | MT769216 | MT769162 | This study |
|  | ZMB 30712-1 | MT769217 | MT769163 | This study |
|  | ZMB 30712-2 | MT769218 | MT769164 | This study |
| *Caridina marlenae* | ZMB 29519-1 |  | MT782050 | This study |
|  | ZMB 29519-2 |  | MT782051 | This study |
|  | ZMB 29519-10 | MT769219 | MT769165 | This study |
|  | ZMB 29519-11 | MT769220 | MT769166 | This study |
|  | ZMB 29519-12 | MT769221 | MT769167 | This study |
|  | ZMB 30199-1 | MT769222 |  | This study |
|  | ZMB 30199-2 | MT769223 | MT769168 | This study |
| *Caridina mayamareenae* | ZMB 29619-1 |  | MT769169 | This study |
|  | ZMB 29627-1 | MT769224 | MT769170 | This study |
|  | ZMB 30388-1 | MT769225 | MT769191 | This study |
|  | ZMB 30709-1 | MT769226 | MT769171 | This study |
|  | ZMB 30709-2 | MT769227 | MT769172 | This study |
|  | ZMB 30710-1 |  | MT769173 | This study |
|  | ZMB 30710-2 | MT769228 | MT769174 | This study |
|  | ZMB 30711-1 | MT769229 | MT769175 | This study |
|  | ZMB 30711-2 | MT769230 | MT769176 | This study |
|  | ZMB 30714-1 | MT769231 | MT769177 | This study |
|  | ZMB 30714-2 | MT769232 | MT769178 | This study |
| *Caridina poso* | ZMB 28063-1 | MT769234 | MT769180 | This study |
|  | ZMB 28063-2 | MT769235 |  | This study |
|  | ZMB 29621-1 | MT769236 | MT769181 | This study |
|  | ZMB 29621-2 | MT769237 | MT769182 | This study |
|  | ZMB 29624-1 | MT769238 | MT769183 | This study |
| *Caridina sarasinorum* | ZMB 28056-1 | MT769239 | MT769184 | This study |
|  | ZMB 28057-1 | MT769240 | MT769185 | This study |
|  | ZMB 28057-2 | MT769241 | MT769186 | This study |
|  | ZMB 28060-1 | MT769242 | MT769187 | This study |
|  | ZMB 28060-2 | MT769243 | MT769188 | This study |
|  | ZMB 29068-1 | AM747759 | AM747687 | von Rintelen et al. 2007 |
|  | ZMB 29137-1 | AM747760 | AM747690 | von Rintelen et al. 2007 |
|  | ZMB 29201a-1 | AM747761 | AM747688 | von Rintelen et al. 2007 |
|  | ZMB 29201b-1 | AM747762 | AM747689 | von Rintelen et al. 2007 |
|  | ZMB 29261-1 | AM747763 | AM747679 | von Rintelen et al. 2007 |
|  | ZMB 29288-1 | AM747764 | AM747680 | von Rintelen et al. 2007 |
|  | ZMB 29383-1 | AM747765 | AM747681 | von Rintelen et al. 2007 |
|  | ZMB 29386-1 | AM747766 | AM747682 | von Rintelen et al. 2007 |
|  | ZMB 29388-1 | AM747767 | AM747683 | von Rintelen et al. 2007 |
|  | ZMB 29402-1 | AM747768 | AM747684 | von Rintelen et al. 2007 |
|  | ZMB 29403-1 | AM747769 | AM747685 | von Rintelen et al. 2007 |
|  | ZMB 29406-1 | AM747770 | AM747686 | von Rintelen et al. 2007 |
|  | ZMB 30224-1 | MT769244 | MT769189 | This study |
|  | ZMB 30224-2 | MT769245 | MT769190 | This study |
| *Caridina schenkeli* | ZMB 29159-1 | AM747774 | AM747699 | von Rintelen et al. 2007 |
|  | ZMB 29159-2 | AM747775 | AM747700 | von Rintelen et al. 2007 |
|  | ZMB 29254-1 | AM747778 | AM747710 | von Rintelen et al. 2007 |
|  | ZMB 29407-2 | AM747789 | AM747701 | von Rintelen et al. 2007 |
|  | ZMB 29441-1 | AM747792 | AM747705 | von Rintelen et al. 2007 |
|  | ZMB 29441-2 | AM747793 | AM747706 | von Rintelen et al. 2007 |
|  | ZMB 29442-1 | AM747794 | AM747702 | von Rintelen et al. 2007 |
|  | ZMB 29442-2 | AM747795 | AM747703 | von Rintelen et al. 2007 |
|  | ZMB 29443-1 | AM747796 | AM747704 | von Rintelen et al. 2007 |
|  | ZMB 29444-1 | AM747797 | AM747697 | von Rintelen et al. 2007 |
|  | ZMB 29444-2 | AM747798 | AM747698 | von Rintelen et al. 2007 |
|  | ZMB 29445-1 | AM747799 | AM747711 | von Rintelen et al. 2007 |
|  | ZMB 29445-2 | AM747800 | AM747712 | von Rintelen et al. 2007 |
|  | ZMB 29446-1 | AM747801 | AM747708 | von Rintelen et al. 2007 |
|  | ZMB 29446-2 | AM747802 | AM747709 | von Rintelen et al. 2007 |
|  | ZMB 29457-1 | AM747804 | AM747707 | von Rintelen et al. 2007 |
| **Outgroup taxa from Sulawesi** | | | |  |
| *Caridina lanceolata* | ZMB 29082-1 | FM201797 | FM201919 | von Rintelen et al. 2010 |
| *C. mahalona* | ZMB 29192-1 | FM201860 | FM202002 | von Rintelen et al. 2010 |
| *C. opaensis* | ZMB 29008-1 | MT769233 | MT769179 | This study |

*von Rintelen K, von Rintelen T, Glaubrecht M (2007) Molecular phylogeny and diversification of freshwater shrimps (Decapoda, Atyidae, Caridina) from ancient Lake Poso (Sulawesi, Indonesia) — the importance of being colourful. Molecular Phylogenetics and Evolution, 45: 1033–1041.

von Rintelen K, Glaubrecht M, Schubart CD, Wessel A, von Rintelen T (2010) Adaptive radiation and ecological diversification of Sulwesi’s ancient lake shrimps. Evolution, 64: 3287–99.
