# Supplementary material for: Lake Poso's shrimp fauna revisited: the description of five new species of the genus Caridina (Crustacea, Decapoda, Atyidae) more than doubles the number of endemic lacustrine species
Source: Zookeys. 2021 Jan 4;1009:81–122. doi: 10.3897/zookeys.1009.54303 (PMC7801368; doi:10.3897/zookeys.1009.54303)
Supplement: Supplementary material 2 — Supplementary figures [file zookeys-1009-081-s002.docx]

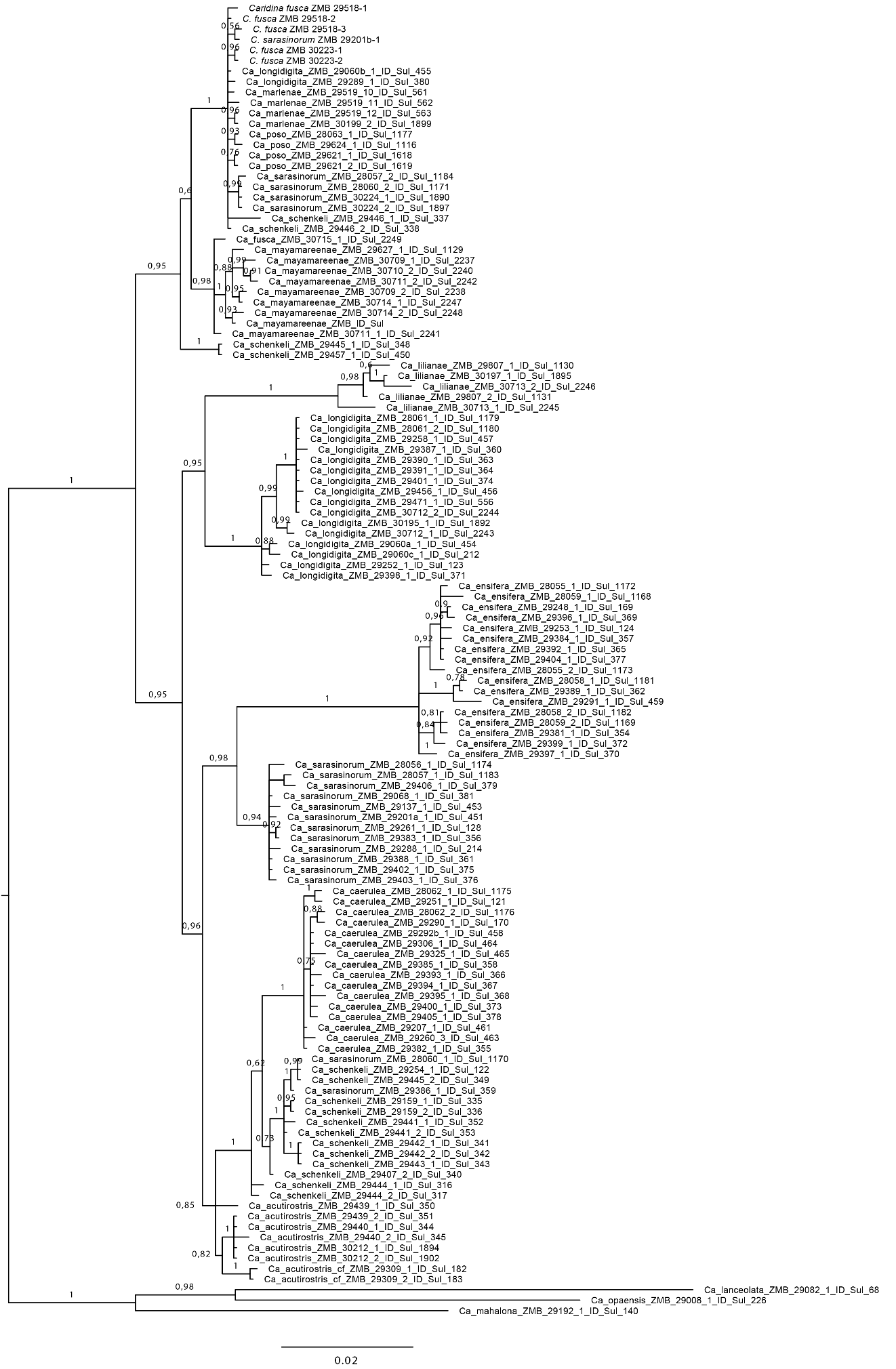


Supplementary Figure 1. Phylogenetic relationships reconstructed by BI analyses of two mitochondrial gene fragments (topology based on concatenated 16S and COI datasets). The scale bar indicates the substitution rate. Original Bayesian posterior probabilities of Fig. 13.


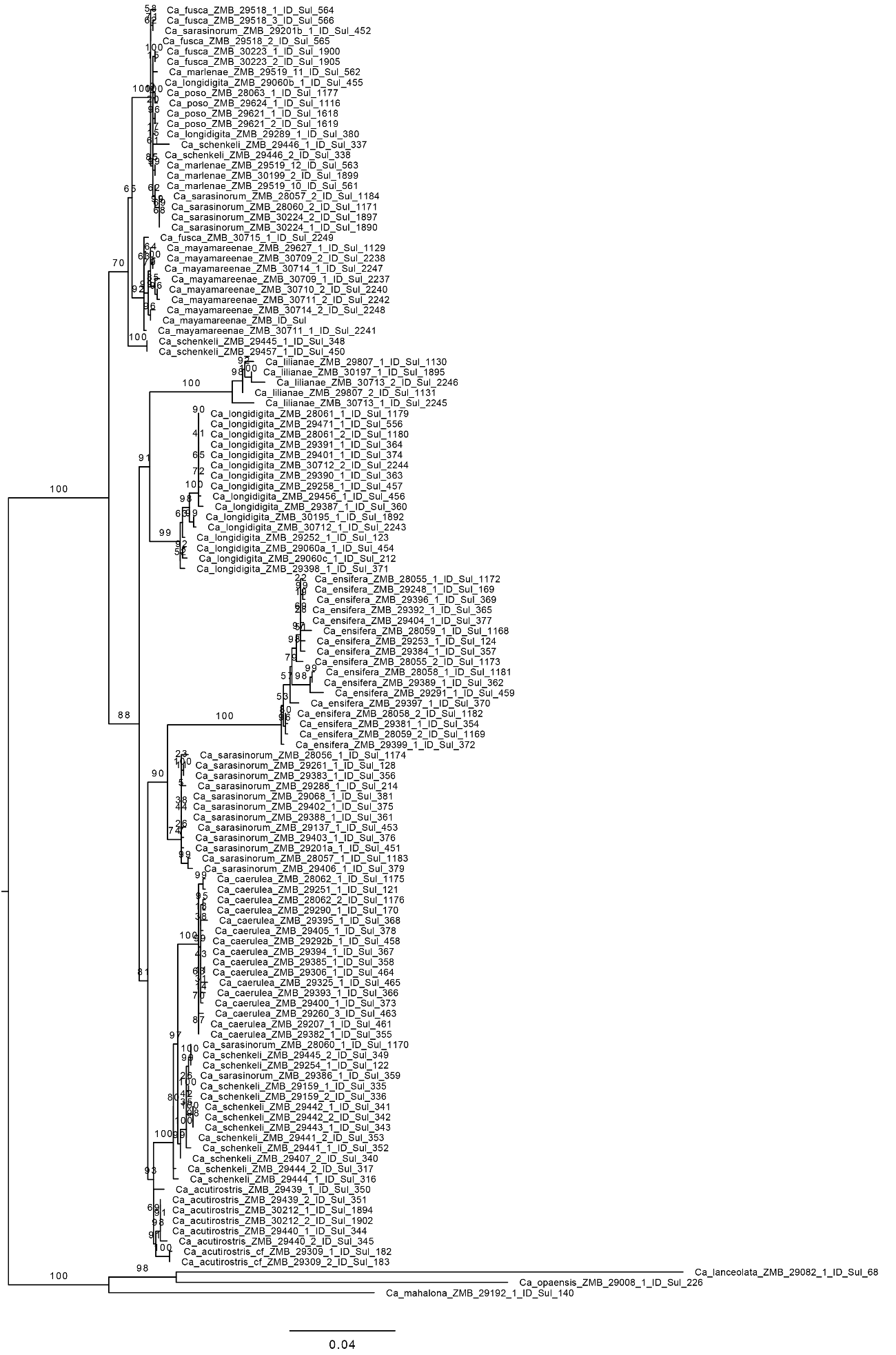


Supplementary Figure 2. Phylogenetic relationships reconstructed by ML analyses of two mitochondrial gene fragments (topology based on concatenated 16S and COI datasets). The scale bar indicates the substitution rate.


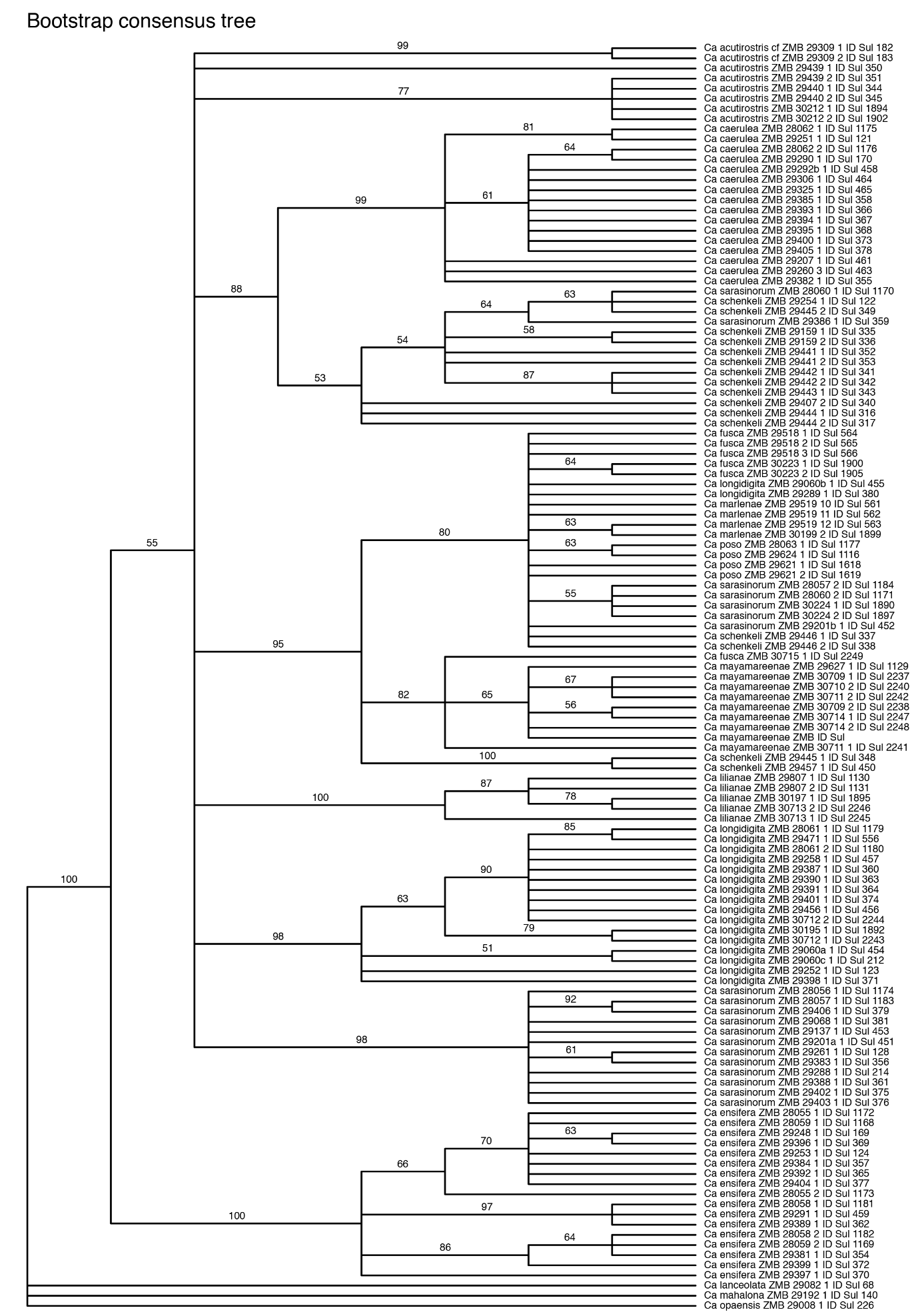


Supplementary Figure 3. Phylogenetic relationships reconstructed by MP analyses of two mitochondrial gene fragments (topology based on concatenated 16S and COI datasets).


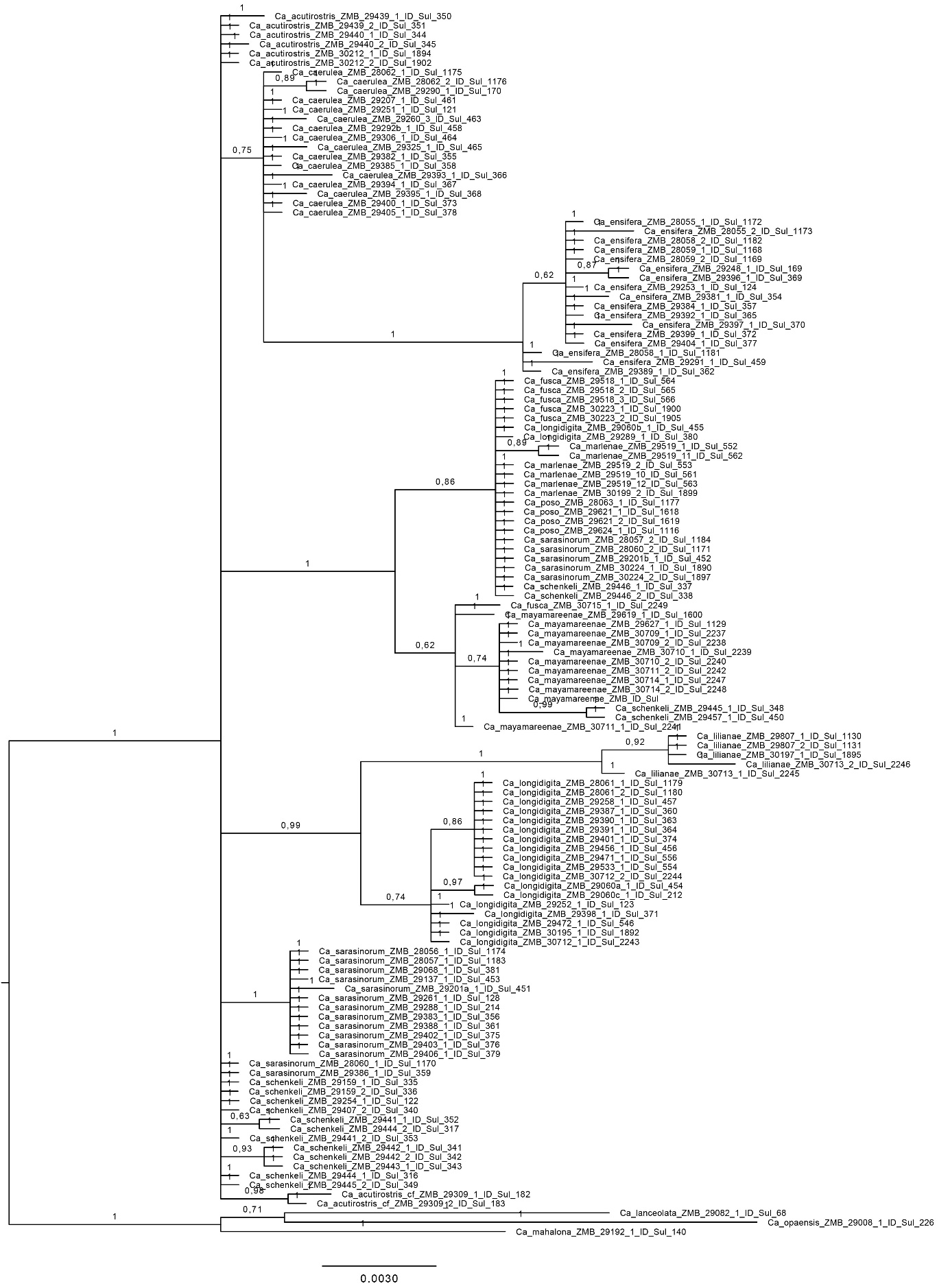


Supplementary Figure 4. Phylogenetic relationships reconstructed by ML analyses of one mitochondrial gene fragments (16S). The scale bar indicates the substitution rate.


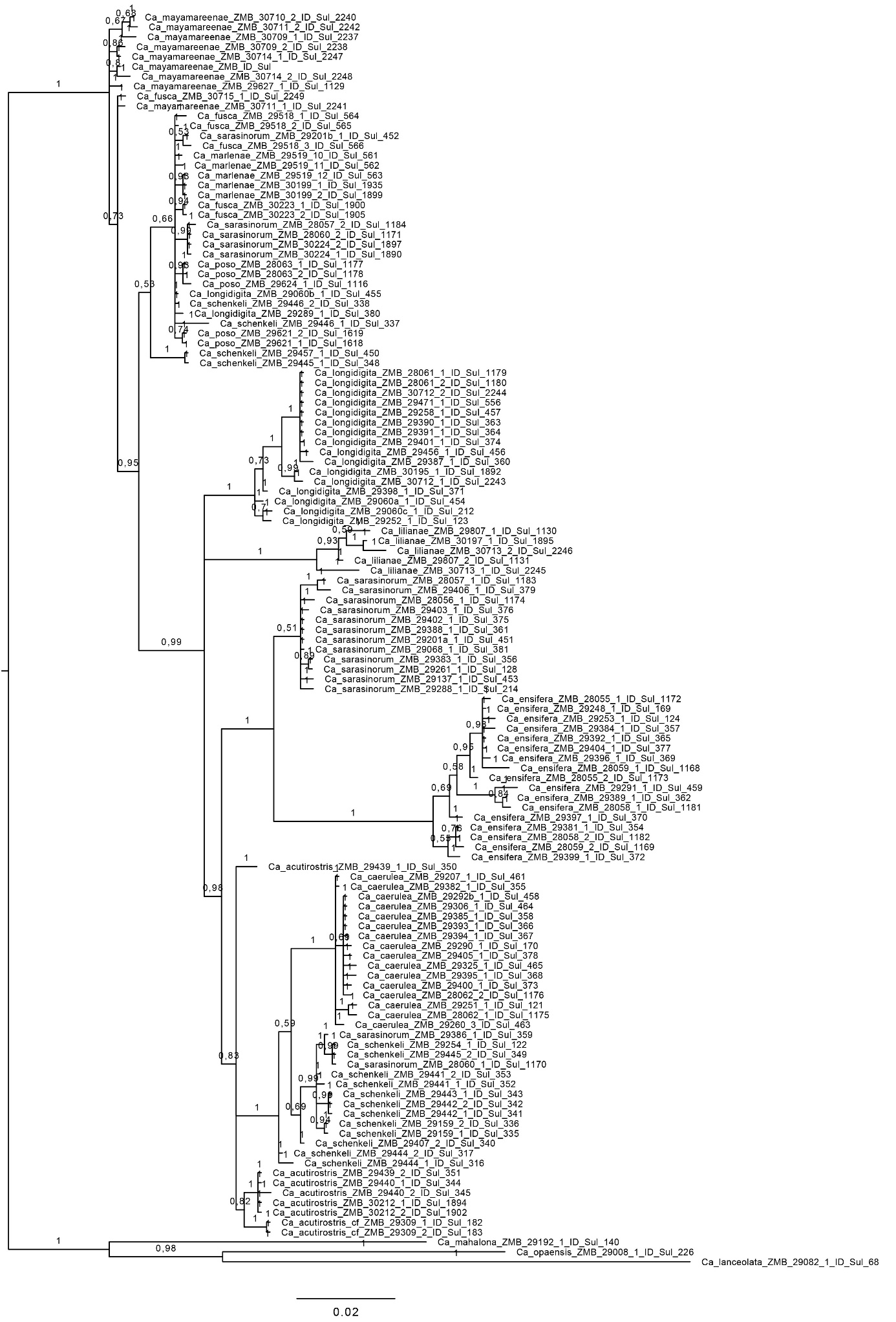


Supplementary Figure 5. Phylogenetic relationships reconstructed by ML analyses of one mitochondrial gene fragments (COI). The scale bar indicates the substitution rate.
